# Strategic targeting of Cas9 nickase induces large segmental duplications

**DOI:** 10.1016/j.xgen.2024.100610

**Published:** 2024-07-24

**Authors:** Yuki Sugiyama, Satoshi Okada, Yasukazu Daigaku, Emiko Kusumoto, Takashi Ito

**Affiliations:** 1 Department of Biochemistry, Kyushu University Graduate School of Medical Sciences, Fukuoka 812-8582, Japan; 2 Cancer Genome Dynamics Project, Cancer Institute, Japanese Foundation for Cancer Research, Tokyo 135-8550, Japan

## Abstract

Gene/segmental duplications play crucial roles in genome evolution and variation. Here, we introduce paired nicking-induced amplification (PNAmp) for their experimental induction. PNAmp strategically places two Cas9 nickases upstream and downstream of a replication origin on opposite strands. This configuration directs the sister replication forks initiated from the origin to break at the nicks, generating a pair of one-ended double-strand breaks. If homologous sequences flank the two break sites, then end resection converts them to single-stranded DNAs that readily anneal to drive duplication of the region bounded by the homologous sequences. PNAmp induces duplication of segments as large as ∼1 Mb with efficiencies exceeding 10% in the budding yeast *Saccharomyces cerevisiae*. Furthermore, appropriate splint DNAs allow PNAmp to duplicate/multiplicate even segments not bounded by homologous sequences. We also provide evidence for PNAmp in mammalian cells. Therefore, PNAmp provides a prototype method to induce structural variations by manipulating replication fork progression.

## Highlights

• PNAmp uses paired nicking to duplicate segments bounded by direct repeats
• PNAmp can efficiently duplicate even megabase-sized segments in budding yeast
• Splint DNA allows PNAmp to duplicate even segments not bounded by direct repeats
• PNAmp is applicable to mammalian cells

## Graphical abstract

**Figure:**
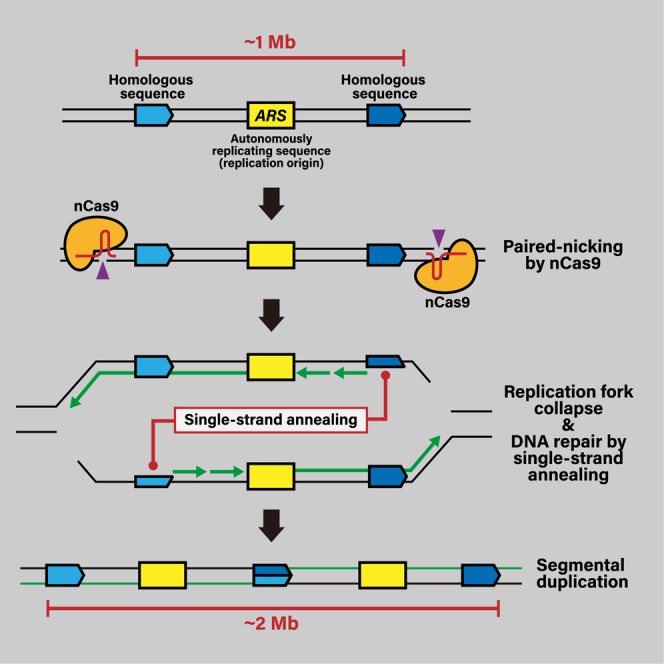

## Teaser

Sugiyama et al. describe PNAmp as an efficient method to induce large segmental duplications by strategically targeting two Cas9 nickases to the upstream and downstream of a repeat-bounded target region. PNAmp represents a distinct class of genome editing methods that disrupt replication fork progression to induce structural variations.

## Introduction

Gene duplication is a critical driver of evolution.^1^ This notion has been increasingly strengthened by the wealth of comparative genomics data.^2^ Duplicated genes contribute to evolution through dosage amplification and functional differentiation (sub- and neo-functionalization).^1^^,^^2^^,^^3^ Although most duplicated copies eventually lose their protein-coding capacity to become pseudogenes, some of them may still exert their functions through DNA- or RNA-based mechanisms to contribute to evolution.^4^ The advent of personal genomics has revealed the extent of genetic diversity in human populations: a single person likely carries ∼5 million single-nucleotide variations, ∼600,000 insertions/deletion variations, and ∼25,000 structural variations (SVs), compared to a reference human genome.^5^^,^^6^^,^^7^^,^^8^ Since SVs have larger sizes than the other two types of genomic variations, they account for three-fourths of the variable portion of a human genome sequence.^5^ These SVs include duplications and deletions resulting in copy-number variations, some of which occur in germline and somatic cells to participate in the pathogenesis of various sporadic diseases and cancers, respectively.^9^^,^^10^^,^^11^^,^^12^ More recently, long-read sequencing technologies have enabled the complete deciphering of complex SVs, leading to the telomere-to-telomere genome assembly^13^ and the pan-genome analysis^14^ in humans. Although segmental duplications (SDs) have long remained untouched regions of the human genome, a thorough analysis has uncovered their unique nature, including increased mutation and gene conversion.^15^

While the structural analysis of gene duplications and SDs has improved remarkably, the mechanisms for their emergence have remained largely speculative. In addition to the whole-genome duplication, retrotransposition and non-allelic homologous recombination (NAHR) are the two main mechanisms postulated to have contributed to gene duplication.^2^ Retrotransposition occurs on a gene-by-gene basis to disperse processed copies of a single gene. The most successful examples of retrotransposition are the retrotransposons scattered throughout the genome, which often serve as drivers to trigger NAHR. Experimental systems to mobilize retrotransposons have deepened the mechanistic understanding of retrotransposition.^16^

In contrast to the gene-by-gene nature of retrotransposition, NAHR can involve two or more genes to induce large tandem SDs. Alternatively, it can include only a subset of exons of a gene to generate its variants encoding proteins with altered domain architecture. In addition, it can iterate to induce tandem multiplications, forming loci composed of large numbers of paralogs, such as those encoding olfactory receptors and immunoglobulins. NAHR relies on direct repeats that recombine with one another through either unequal crossing-over or break-induced replication (BIR).^17^ A two-ended double-strand break (DSB) occurring in one of the direct repeats initiates crossing-over. In contrast, a one-ended DSB, generated by the collapse of a replication fork passing through one of the direct repeats, initiates BIR. Despite the highly innovative role of tandem duplications in evolution, it remains challenging to induce them experimentally.

In this context, it is noteworthy that a previous study reported an intriguing phenomenon, re-replication-induced gene amplification (RRIGA), which efficiently induces large SDs in the budding yeast *Saccharomyces cerevisiae*.^17^^,^^18^ DNA replication occurs once and never twice in the cell cycle because eukaryotic cells have evolved the system to prevent re-initiation. Genetic ablation of this system in the budding yeast derepresses re-replication initiated from a specific replication origin, or autonomously replicating sequence 317 (*ARS317*), in the G2/M phase.^19^ Since the re-replication forks are prone to breakage,^17^ the sister forks initiated from *ARS317* may collapse simultaneously to generate a pair of one-ended DSBs, with each sister chromatid carrying a single break. If retrotransposon Ty (transposon yeast) elements are present in the same orientation in the re-replicated region, then subsequent end resection will expose the top and bottom strands of the Ty elements as single-stranded DNA (ssDNA). These ssDNA strands will readily anneal to one another to drive duplication of the region bounded by the two Ty elements. RRIGA is a unique form of NAHR because it depends on single-strand annealing (SSA) but not unequal crossing-over or BIR and is experimentally inducible. Requiring three mutant alleles (*orc6-S116A*, *MCM7-2NLS*, *pGAL1-*Δ*nt-cdc6-cdk2A*) and the specific replication origin *ARS317*, RRIGA should have barely occurred in the natural context. However, if a similar mechanism operates upon the collapse of S phase replication forks initiated by any ARS, then it would have generated many more SDs than RRIGA, thus having played a more critical role in evolution.

We hypothesized that a Cas9-based trick could induce SDs, as we previously showed that catalytically inactive Cas9 (dCas9 [dead Cas9]) impairs DNA replication fork progression to induce focal genomic instability, leading to copy-number alterations in the *CUP1* tandem array in the budding yeast genome.^20^ This result suggests that dCas9 provides a versatile tool to induce replication fork stalling and subsequent collapse at virtually any site in the genome *in vivo*, leading to the generation of one-ended DSB. Interestingly, a single-molecule observation study showed that the replisome disassembles upon collision with Cas9 nickase (nCas9) *in vitro*.^21^ Thus, we assume that nCas9 can be a more direct inducer of replication fork collapse *in vivo* than dCas9. Based on these considerations, we have conceived a strategy to induce SDs via nCas9-mediated paired nicking.

## Design

Our strategy involves positioning two nCas9s upstream and downstream of a replication origin. One nCas9 targets the top strand, while the other targets the bottom strand. As a result, the two replication forks initiated from the origin collide with the nCas9s, creating a pair of one-ended DSBs. These breaks become single stranded as the cell is in the S phase, during which DSBs are actively end-resected. If identical sequences flank the two DSBs in the same orientation to form direct repeats, then the top and bottom strands of the repeats are exposed as ssDNA. Mutual annealing between these ssDNAs should drive the duplication of the region bounded by the direct repeats.

## Results

### Paired nicking duplicates a genomic segment bounded by direct repeats

**Figure 1:**
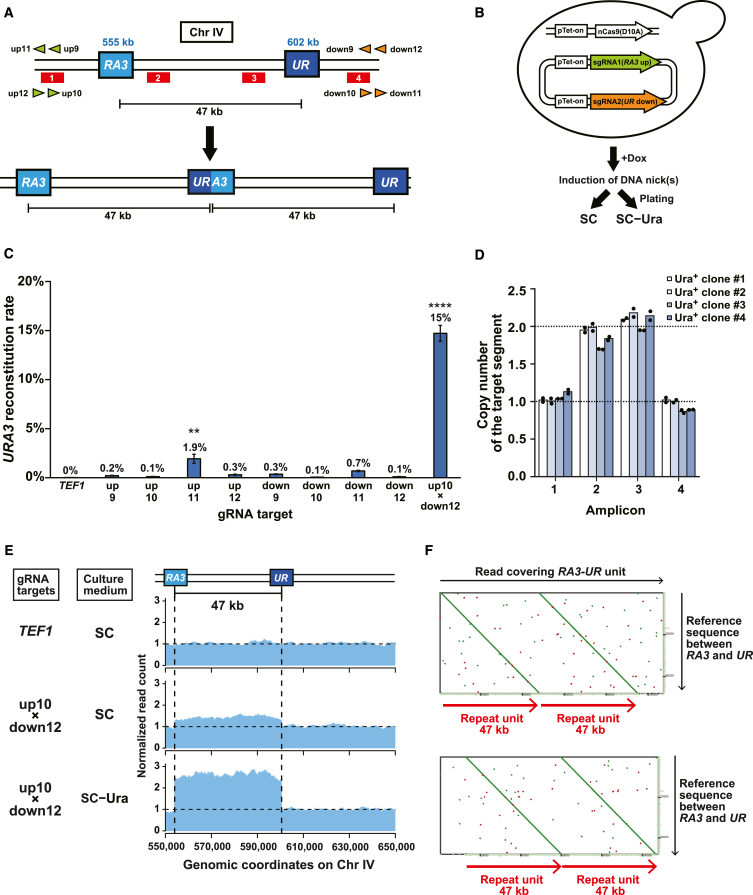
Paired nicking duplicates a genomic segment bounded by direct repeats (A) Genetic reporter to detect tandem duplication of a 47-kb segment on chromosome IV. Blue number, genomic coordinate; arrowhead, gRNA target sequences; red bar, qPCR amplicon used in (D). (B) Schematic of the genetic reporter assay for target duplication. (C) *URA3* reconstitution rates of strains expressing various gRNAs. Error bar, SEM (*n* = 3). Statistical significance between the sample strains and the control strain expressing *TEF1* gRNA was examined using Dunnett’s test (∗∗*p* < 0.01; ∗∗∗*p* < 0.001). (D) Copy number of the target segment in 4 Ura^+^ clones measured with qPCR. The positions of the qPCR amplicons are shown in (A). Dots indicate technical replicates for each clone. (E) Normalized read count of the 47-kb segment in whole-genome nanopore sequencing. Pooled colonies from the indicated media were used for sequencing. (F) Representative dot plots between nanopore reads and the 47-kb segment. We used Ura^+^ clone 1 in (D). Of the 28,013 reads over 50 kb, 4 covered the entire duplicon.

We constructed a genetic reporter strain of the budding yeast *S. cerevisiae* to evaluate whether and how efficiently nCas9 can induce the duplication of a genomic segment bounded by identical sequences in the same orientation or direct repeats. In this strain, chromosome IV carries two DNA fragments derived from the *URA3* gene, namely *RA3* and *UR*, which share a 391-bp sequence *R*, at the 555 and 602 kb positions, respectively (Figure 1A; see method details for the customized reference genome sequence). The two *R*s serve as direct repeats to delineate the 47-kb target segment. If a homology-dependent DNA repair event occurs between the direct repeats, the 47-kb segment should duplicate in a head-to-tail manner to reconstitute the *URA3* gene, thereby rendering the cells viable on a synthetic complete medium without uracil (SC−Ura). To induce a repair event, we used appropriate guide RNAs (gRNAs) to recruit nCas9 (D10A), individually or simultaneously, to positions upstream of *RA3* and downstream of *UR* (Figures S1A–S1C). Following the expression of nCas9 and gRNAs by the Tet-on system, we spread the cells on SC and SC−Ura agar plates to calculate the *URA3* reconstitution rate from the colony numbers (Figure 1B).

With a gRNA targeting a control position on another chromosome (*TEF1* on chromosome XVI), the *URA3* reconstitution did not occur at a frequency detectable in this assay. With single gRNAs, each targeting either an upstream position of *RA3* or a downstream position of *UR*, the *URA3* reconstitution occurred with an efficiency of <2% (Figure 1C). These gRNAs remained ineffective even when paired with the *TEF1* gRNA (Figure S1D). In contrast, a pair of gRNAs simultaneously targeting the upstream and downstream flanking positions, one on the top strand and the other on the bottom strand, induced the reconstitution much more efficiently than individual single gRNAs (15% vs. 0.1% and 0.1%) (Figure 1C).

To confirm the duplication of the 47-kb target segment in the Ura^+^ clones, we designed four qPCR amplicons between and outside *RA3* and *UR* (Figure 1A). As expected, the copy number increased only at the amplicons designed between *RA3* and *UR* (Figure 1D, amplicons 2 and 3) but not at those outside the target segment (Figure 1D, amplicons 1 and 4). We next performed whole-genome sequencing on pooled Ura^+^ colonies using the Oxford Nanopore MinION sequencer and mapped the reads to the reference genome. The normalized read count showed an approximately 2-fold increase throughout the 47-kb segment, suggesting its duplication (Figure 1E). Finally, we took advantage of the long read length of the data to identify such reads that included both the upstream and downstream flanking regions of *RA3* and *UR*, respectively. Dot plots between such reads and the reference sequence of the 47-kb segment provided direct, unambiguous evidence for the expected tandem duplication (Figure 1F).

Therefore, strategic targeting of nCas9 can efficiently induce the duplication of a segment bounded by direct repeats. We called this method paired nicking-induced amplification (PNAmp).

### SSA mediates PNAmp

**Figure 2:**
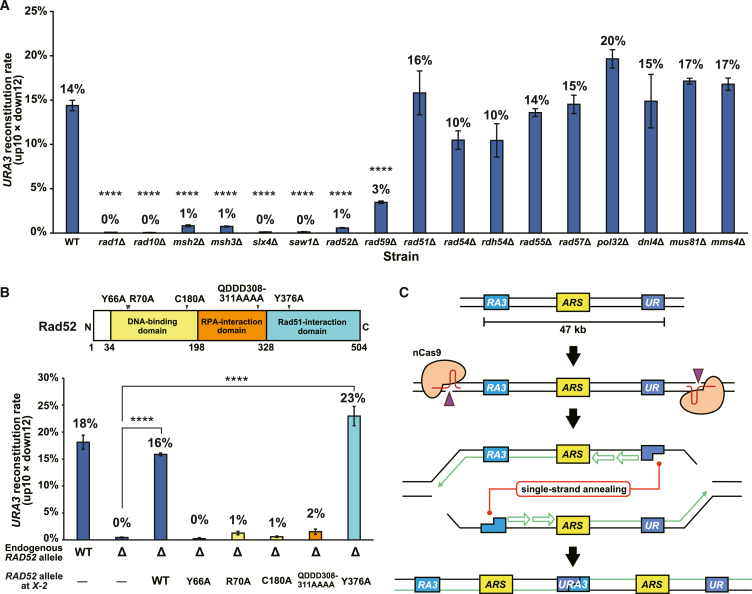
SSA mediates PNAmp (A) Efficiency of PNAmp in strains deleted for DNA repair genes. Error bar, SEM (*n* = 3). Statistical significance between the mutant strains and the WT strain was examined using Dunnett’s test (∗∗∗∗*p* < 0.0001). (B) Effects of separation-of-function *rad52* alleles. Top, Rad52 structure with the positions of domain boundaries and amino acid substitutions in *rad52* alleles. Bottom, PNAmp efficiency in the *rad52*Δ strain, with each allele expressed from the *X-2* locus by the *CUP2* promoter. Error bar, SEM (*n* = 3). Statistical significance was examined using Dunnett’s test (∗∗∗∗*p* < 0.0001). (C) Mechanistic model of PNAmp.

To gain mechanistic insight into PNAmp, we examined its efficiency in a series of strains deleted for genes involved in DNA repair. The efficiency of PNAmp showed a drastic decrease in *rad1*Δ, *rad10*Δ, *msh2*Δ, *msh3*Δ, *slx4*Δ, and *saw1*Δ cells (Figure 2A). These results indicated that genes involved in flap cleavage are essential for PNAmp.^22^^,^^23^^,^^24^^,^^25^^,^^26^^,^^27^ Furthermore, the deletion of *RAD52* and its paralog *RAD59* strongly suppressed PNAmp (Figure 2A), suggesting the involvement of DNA annealing activity.^28^^,^^29^^,^^30^ In contrast, the deletion of genes required for homology-mediated strand invasion (*RAD51*, *RAD54*, and *RAD55)*,^31^ BIR (*POL32*),^32^ non-homologous end joining (*DNL4*),^33^ and Holliday junction resolution (*MUS81* and *MMS4*)^34^^,^^35^ did not affect the *URA3* reconstitution rate (Figure 2A). These data collectively suggest that PNAmp is dependent on SSA.^36^^,^^37^

Rad52 consists of N-terminal, central, and C-terminal domains. To investigate which domain mainly contributes to PNAmp, we used separation-of-function *rad52* alleles (Figure 2B, top). The N-terminal domain is evolutionarily conserved among eukaryotes and can bind ssDNA to stimulate its annealing.^38^ Amino acid substitutions in this domain (Y66A, R70A, or C180A) disrupt the SSA activity.^38^^,^^39^^,^^40^ The central domain binds the ssDNA-binding protein RPA.^41^ Amino acid substitutions in this domain (QDDD308–311AAAA) render the protein defective in RPA binding and, hence, the recruitment to DSB sites.^41^ The C-terminal domain has the mediator activity to recruit Rad51 to RPA-coated ssDNA. An amino acid substitution in this domain (Y376A) renders the protein unable to bind Rad51.^42^^,^^43^

We integrated the wild-type (WT) *RAD52* or one of the separation-of-function *rad52* alleles under the control of the *CUP2* promoter into a safe harbor locus on chromosome X in a *rad52*Δ strain. All these strains except the *rad52-C180A* strain, expressed the WT and mutant Rad52 proteins at comparable levels (Figure S2A). The *rad52-Y66A*, *rad52-R70A*, *rad52-C180A*, and *rad52-QDDD308–311AAAA* alleles did not suppress the PNAmp defect, whereas the *rad52-Y376A* allele did (Figure 2B, bottom), reinforcing the notion that SSA activity is critical for PNAmp. These results were consistent with the observations that PNAmp is proficient and defective in the absence of *RAD51* and *RAD59*, respectively, the latter encoding the Rad52 paralog carrying only the N-terminal domain to support SSA (Figure 2A).

Based on these results, we proposed a model for PNAmp (Figure 2C). In this model, two replication forks progress from the inside to the outside of the target segment and encounter the nicks generated by nCas9, leading to replisome disassembly and the generation of a pair of one-ended DSBs, one upstream and the other downstream of the direct repeats. These DSBs undergo end resection to generate 3′ protruding ssDNAs, one containing the top strand and the other containing the bottom strand of the direct repeats. Rad52 anneals these strands with the aid of Rad59, and the Rad1–Rad10 complex removes non-homologous flaps. Subsequent sealing of the nicks completes the duplication of the segment bounded by the direct repeats (Figure 2C).

### PNAmp occurs by both *trans*- and *cis*-nicking

To investigate whether PNAmp occurs using gRNA pairs other than the one used above (Figure 1C), we tested four gRNA pairs for their ability to induce the *URA3* reconstitution in the reporter strain (Figure S2B). Based on the proposed model, all these gRNA pairs introduce one nick in the top strand and the other in the bottom strand (*trans*-nicking). All four gRNA pairs induced PNAmp with efficiencies ranging from 3% to 35% (Figure S2B), demonstrating that PNAmp is not specific to a particular gRNA pair.

We also tested four other gRNA pairs that induce both nicks on the same strand (*cis*-nicking). Unexpectedly, Ura^+^ colonies appeared upon *cis*-nicking, with efficiencies ranging from 5% to 21% (Figure S2B). Therefore, we used the same series of mutants tested for “canonical” PNAmp with *trans*-nicking (Figure 2A) to gain insight into the mechanism of “non-canonical” PNAmp with *cis*-nicking. To our interest, non-canonical PNAmp depends not only on the genes required for canonical PNAmp but also on those involved in homology search and strand invasion (*RAD51*, *RAD54*, *RAD55*, and *RAD57*) (Figure S2C). These results suggest that the non-canonical PNAmp has a different mechanism from the canonical PNAmp, which should await future studies. In this study, we focused on the canonical PNAmp using *trans*-nicking.

### Replication initiated from within the target segment enhances PNAmp

**Figure 3:**
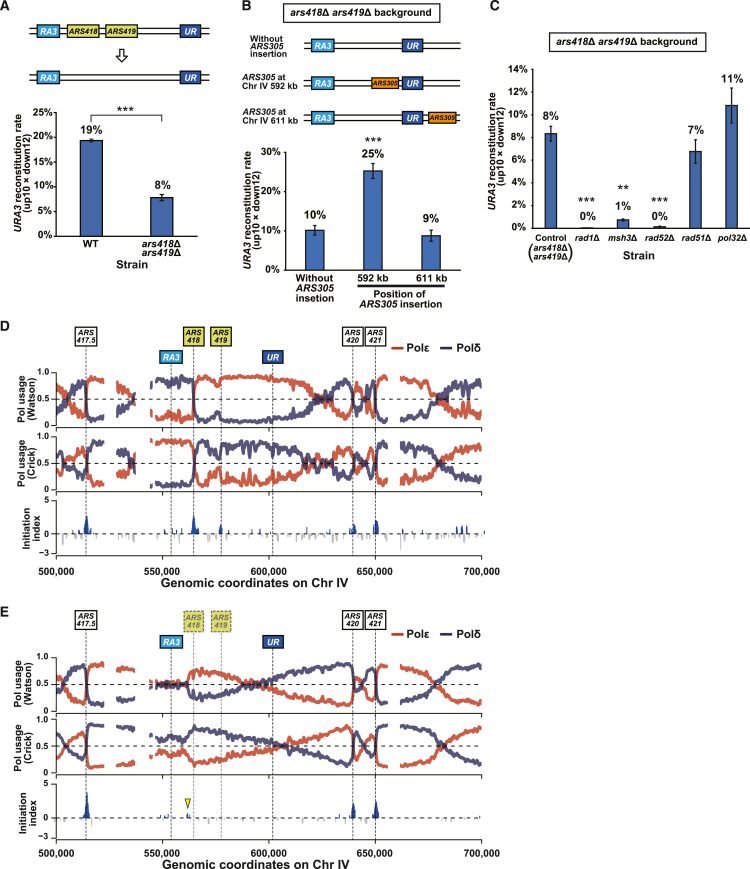
Replication initiated from within the target segment enhances PNAmp (A) PNAmp in the WT and *ars418*Δ *ars419*Δ strains. Error bar, SEM (*n* = 3). Statistical significance was examined using Student’s t test (∗∗∗*p* < 0.001). (B) Effect of *ARS305* insertion on PNAmp in the *ars418*Δ *ars419*Δ strain. Error bar, SEM (*n* = 3). Statistical significance between the strains without and with *ARS305* was examined using Dunnett’s test (∗∗∗*p* < 0.001). (C) Effect of deleting DNA repair genes on PNAmp in the *ars418*Δ *ars419*Δ strain. Error bar, SEM (*n* = 3). Statistical significance between the control and mutant strains was examined using Dunnett’s test (∗∗*p* < 0.01; ∗∗∗*p* < 0.001). (D and E) Pu-seq patterns around the 47-kb target segment in the WT strain (D) and the *ars418*Δ *ars419*Δ strain (E). The yellow triangle in (E) indicates the region showing a weak ARS activity. The interruptions in the plots are due to the masking of Ty elements and *ENA1/ENA2/ENA5* tandem array.

Our model postulates that two replication forks initiated from within the target segment play a critical role in PNAmp (Figure 2C). Therefore, we examined the efficiency of PNAmp in a strain deleted for the ARSs annotated in the 47-kb segment (*ARS418* and *ARS419*) (Figure 3A, top). As expected, PNAmp efficiency was significantly reduced in the *ars418*Δ *ars419*Δ strain, demonstrating the importance of replication initiated from within the target segment (Figure 3A, bottom). Conversely, we tested whether the insertion of an exogenous ARS restores the efficiency of PNAmp by integrating *ARS305*, an efficient early-firing ARS,^39^ inside and outside the target segment in the *ars418*Δ *ars419*Δ strain (Figure 3B, top). The strain with *ARS305* insertion in the target segment showed a significantly higher PNAmp efficiency than the parental *ars418*Δ *ars419*Δ strain without the insertion (Figure 3B, bottom). In contrast, there was no difference in efficiency between the strain with *ARS305* insertion outside the segment and the parental *ars418*Δ *ars419*Δ strain (Figure 3B, bottom). These results highlight the critical role of replication initiated from the target segment in efficient PNAmp.

### PNAmp mediates duplication of a segment without annotated ARS

We were intrigued that the *URA3* reconstitution occurred in ∼8% of the surviving colonies of the *ars418*Δ *ars419*Δ strain (Figure 3A, bottom). To gain insight into the mechanism of PNAmp in this strain, we examined the effect of deleting genes involved in DNA repair. The efficiency of PNAmp significantly decreased in the absence of SSA-related genes (*RAD1*, *MSH3*, *RAD52*) but not *RAD51* and *POL32* in the *ars418*Δ *ars419*Δ strain (Figure 3C), as in the WT strain (Figure 2A). These results suggest that the mechanism of PNAmp is identical regardless of the presence or absence of *ARS418* and *ARS419*. One possible scenario is that DNA replication initiates from within the target segment even in the *ars418*Δ *ars419*Δ strain, although less frequently than in the WT strain. Indeed, a previous study showed that unannotated sites around the deleted ARSs initiate DNA replication.^44^

Therefore, we aimed to compare the status of DNA replication in the target segment between the WT and *ars418*Δ *ars419*Δ strains using polymerase usage sequencing (Pu-seq).^45^ This method uses DNA polymerase mutants with enhanced ribonucleotide incorporation to determine the distribution of incorporated ribonucleotides throughout the genome, thereby revealing the division of labor between polymerases. We generated strains harboring *pol2-M644G* and *pol3-L612G* alleles to enhance ribonucleotide incorporation by DNA polymerase ε (Polε) and DNA polymerase δ (Polδ), respectively,^46^^,^^47^ in the background of *rnh201*Δ to protect the incorporated ribonucleotides from removal by RNaseH2. After the alkaline treatment of the genomic DNA to induce cleavage at the ribonucleotides, we prepared sequencing libraries and mapped the obtained reads to the customized reference genome. A reciprocal pattern emerged between the usage of Polε and Polδ on the same strand and between the Watson and Crick strands for each polymerase (Figure S3A), as described previously.^45^

In the target segment, the usage of Polε and Polδ showed a steep reciprocal change at *ARS418*, resulting in a high peak of the initiation index, an indicator of the initiation efficiency calculated from the polymerase usage (Figure 3D). In contrast, *ARS419* showed only a weak reciprocal change and a low initiation index (Figure 3D). These results indicate that *ARS418* is responsible for replicating the target segment most of the time. Notably, the evident reciprocal changes in polymerase usage observed in WT cells disappeared in the *ars418*Δ *ars419*Δ strain, as did the peak of the initiation index (Figure 3E): deletion of the two ARSs resulted in efficient suppression of replication initiated from within the target segment. If initiation does not occur from within the segment at all, then *ARS417.5* and *ARS420* should support the replication of this segment. In this case, the Polε signal on the Watson strand should show a monotonically decreasing rightward slope that starts from *ARS417.5* and ends at *ARS420*. However, this was not the case: the Polε signal slope showed a large concavity around *RA3*, whereas the Polδ signal showed a concomitant convexity (Figure 3E). The Polε and Polδ signals on the Crick strand consistently showed broad convexity and concavity, respectively (Figure 3E). These results indicated the presence of initiation sites around *RA3*, which are intriguingly distributive, in sharp contrast to the focused initiation by the canonical ARS. The initiation index formed a weak peak at 562 kb on chromosome IV of the *ars418*Δ *ars419*Δ strain (Figures 3E and S3B).

To investigate the potential ARS activity of this region, we divided the region containing the initiation index peak into five fragments and individually cloned them to a plasmid carrying a centromere and the *URA3* gene (Figure S3B). We incubated the transformants of these plasmids on SC-Ura agar plates to test whether each fragment could initiate plasmid replication to support cell proliferation (Figure S3C). A strain carrying the plasmid encoding one of the five fragments (fragment 4) showed weak but significant growth compared to the strain carrying an empty plasmid or other fragments, indicating that this fragment has weak ARS activity to replicate the plasmid (Figure S3C).

These results suggest the possibility that DNA replication initiated from the non-canonical, cryptic origins, including the one tested above, supported the PNAmp in the *ars418*Δ *ars419*Δ strain. In addition, a variant form of SSA-mediated mechanism may mediate PNAmp without replication initiated from within the target segment (Figure S4A). Regardless of the underlying mechanisms, PNAmp could amplify a region without annotated ARS, albeit less efficiently. These results indicate that PNAmp may have broader targets than originally thought.

### PNAmp induces tandem duplication of megabase-sized segments

**Figure 4:**
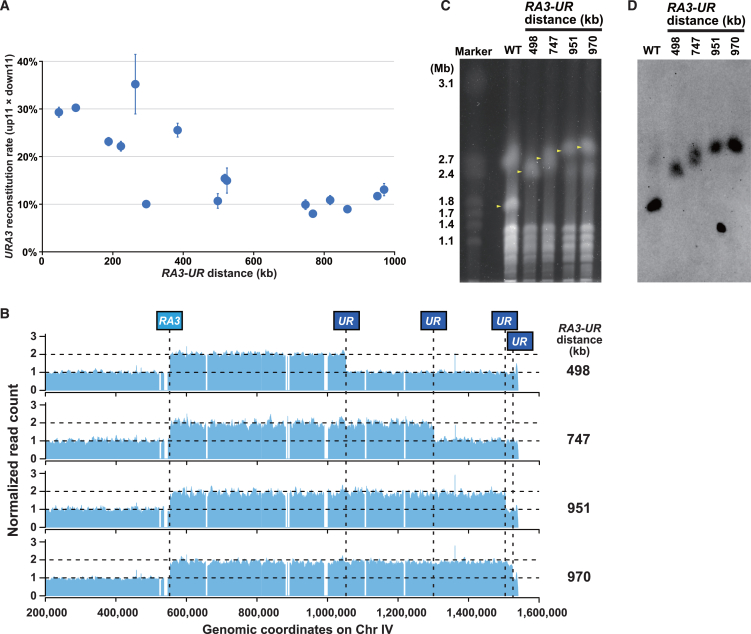
PNAmp induces tandem duplication of megabase-sized segments (A) Effects of the target size on PNAmp. Error bar, SEM (*n* = 3). (B) Normalized read count of the target segments in whole-genome sequencing of 4 representative clones with large target segments, ranging from 498 to 970 kb. The gaps in read count are the repetitive sequences masked in the customized reference genome sequence. (C) PFGE of the clones in (B). Marker *Hansenula wingei* chromosomes; yellow arrowhead, chromosome IV. (D) Southern blot hybridization of the PFGE gel shown in (C) with chromosome IV probes.

We next intended to investigate the maximum size of segments that PNAmp can duplicate. For this purpose, we constructed a series of strains harboring *RA3* and *UR* at different distances. While these strains harbor *RA3* at a fixed position on chromosome IV (555 kb), the position of *UR* and its flanking region containing the gRNA target sequence was variable, resulting in 16 target segments ranging from 47 to 970 kb (Figure 4A). Note that we deleted the gRNA target sequence at its original position (602 kb) to avoid introducing nicks within the target segments. We induced PNAmp in these strains using a pair of gRNAs targeting up11 and down11, which was one of the most effective gRNA pairs for the PNAmp of the 47-kb target segment (Figure S2B). As a result, all 16 strains succeeded in the *URA3* reconstitution with variable efficiency (Figure 4A). Notably, ∼10% or more of the viable colonies were Ura^+^ even when the target segment size reached ∼1 Mb (Figure 4A). To confirm large SDs, we performed whole-genome sequencing of 4 representative strains with 498- to 970-kb target segments. The normalized read count showed a 2-fold increase across the target segments (Figure 4B). In addition, pulsed-field gel electrophoresis (PFGE) revealed an expansion of chromosome IV by the length of the target segment in each strain (Figures 4C and 4D). These results demonstrated that PNAmp induced the expected large SDs.

Although the rate of *URA3* reconstitution tended to decrease as the size of the target segment increased, it was not a simple function of target size (Figure 4A). Because the PNAmp model requires the leftward and rightward forks to replicate *RA3* and *UR*, respectively (Figure 2C), its efficiency is likely to depend on the frequency with which the replication forks from inside the target segment (outward replication forks) arrive at the nicks earlier than the inward replication forks. Therefore, we used the Pu-seq data to calculate the replication fork directionality (RFD) as an indicator of the proportion of replication forks moving to the left or right at each genomic locus (Figure S3D).^48^^,^^49^ Theoretically, ideal target segments for the standard PNAmp have positive RFD values around their *UR* fragments, indicating the dominance of rightward forks. However, PNAmp can also successfully duplicate segments that have *UR* fragments at loci with negative RFD values (Figure S3E). Thus, the combination of target size with RFD value did not explain the efficiency of PNAmp. The PNAmp of segments with undesirable RFD values may include a variant form of SSA-mediated mechanism (Figure S4B). In any case, PNAmp efficiently duplicates even megabase-sized segments, although the determinants of its efficiency are not fully understood.

### PNAmp occurs in natural genomic contexts

We next tested whether PNAmp operates in a natural genomic context, or without using the *URA3* reconstitution reporter, by attempting to duplicate a 105-kb segment bounded by two Ty1 retrotransposable elements on chromosome IV (Figure S5A). After expressing nCas9 and two gRNAs targeting upstream of one Ty1 element (*YDRWTy1-4*) and downstream of the other Ty1 element (*YDRWTy1-5*), we performed whole-genome nanopore sequencing on pooled colonies, as we cannot genetically select cells harboring the intended duplication. The normalized read count showed a 1.15-fold increase in the target segment (Figure S5B, WT). Furthermore, we identified the reads spanning the junction, or a chimera of the two Ty1 elements, connecting the two duplicated copies of the target segment (Figure S5C).

Naturally occurring homologous sequences typically show some level of sequence divergence, which can impact the efficiency of SSA.^50^^,^^51^ The two Ty1 elements used above share 96.3% sequence identity to contain substitutions and insertions or deletions that may adversely affect PNAmp. Intriguingly, deletion of mismatch repair (MMR)-related genes improves the efficiency of SSA between non-identical sequences.^50^^,^^51^ The normalized read count increased to 1.52- to 1.64-fold in strains lacking MMR-related genes (*msh6*Δ, *sgs1*Δ, and *top3*Δ) (Figures S5B and S5D). Moreover, MMR-defective strains, but not their parental strain, even allowed PNAmp of a 24-kb segment bounded by two long terminal repeats sharing only 93% sequence identity (*YDRWdelta25* and *YDRWdelta26*) (Figures S5A and S5E).

These results collectively demonstrate that PNAmp can utilize natural repetitive sequences to induce SD, especially when MMR is compromised.

### PNAmp iterates to multiply target segments

**Figure 5:**
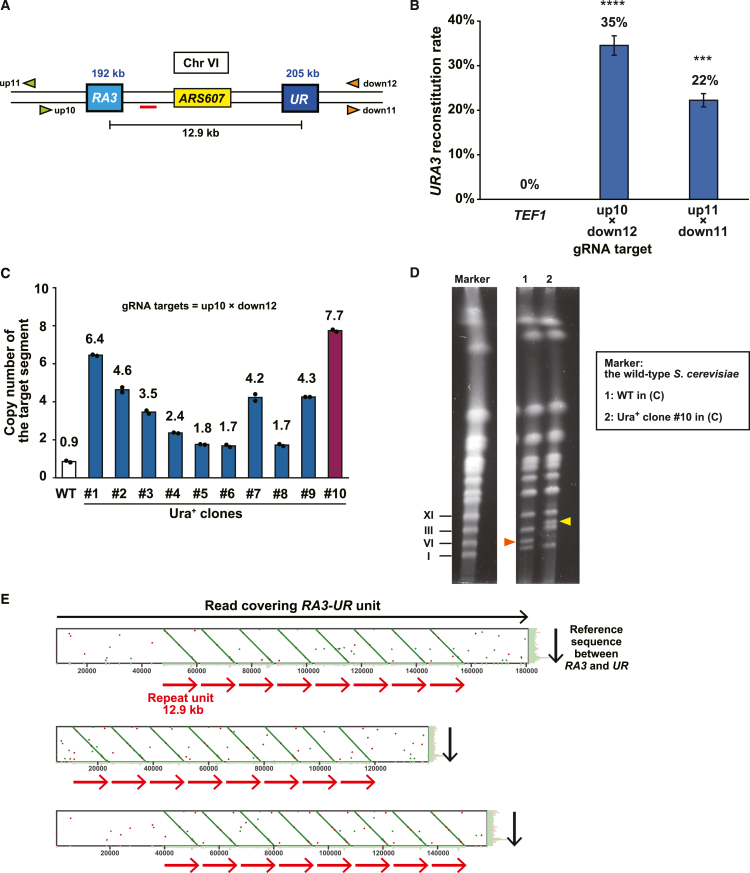
PNAmp iterates to multiply target segments (A) Genetic reporter to detect tandem duplication of a 12.9-kb segment on chromosome VI. Blue number, genomic coordinate; arrowhead, gRNA target; red bar, qPCR amplicon used in (C). (B) *URA3* reconstitution rates of strains expressing various gRNAs. Error bar, SEM (*n* = 3). Statistical significance between the sample strains and the control strain expressing *TEF1* gRNA was examined using Dunnett’s test (∗∗∗*p* < 0.001; ∗∗∗∗*p* < 0.0001). (C) Copy number of the target segment in Ura^+^ clones determined by qPCR. Dots indicate technical replicates for each clone. (D) PFGE of the WT and Ura^+^ clone 10 in (C). Arrowhead, chromosome VI. (E) Dot plots between nanopore reads of the Ura^+^ clone 10 in (C) and the reference sequence of the 12.9-kb segment. Of the 19,236 reads over 100 kb, 21 covered the entire amplicon.

We next intended to duplicate a 12.9-kb segment containing *ARS607* by transplanting the reporter system (Figure 5A). Both gRNA pairs tested successfully induced the reconstitution of the *URA3* gene (Figure 5B). To physically confirm the intended duplication, we examined the copy number of the target segment in the Ura^+^ clones by qPCR. Unexpectedly, the copy numbers varied between ∼2 and ∼8 (Figure 5C). We thus performed PFGE of the clone with the highest copy number in the qPCR assay and found that its chromosome VI was of a size consistent with the presence of seven additional copies of the 12.9-kb segment (Figure 5D). Furthermore, nanopore sequencing of this clone identified reads spanning eight copies of the target segment arrayed in tandem, as indicated by dot plots (Figure 5E). These results suggest that PNAmp occurs iteratively: three cycles of PNAmp should octuplicate the target segment, and subsequent recombinational deletion events would generate cells with different copy numbers. In this context, it is intriguing that normalized read coverage exceeded two copies in the pooled Ura^+^ colonies obtained in the PNAmp of the 47-kb segment on chromosome IV (Figure 1E).

Successive rounds of PNAmp are expected to lead to a progressive increase in the target copy number, which should correlate positively with the duration of nCas9 induction. We observed that extending the induction period resulted in a higher frequency of recovered clones with an elevated target copy number (Figure S6A). These results suggest the potential for regulating copy-number distribution by adjusting the induction period. In addition, it should be possible to select clones with an elevated copy number based on the dosage effect of a gene in the target segment. To test this hypothesis, we integrated an mNeonGreen expression cassette into the target segment (Figure S6B). We observed a positive correlation between the copy number and the fluorescence intensity in the Ura^+^ clones, providing support for this possibility (Figures S6C and S6D). Taken together, iterative PNAmp multiplies target segments, potentially enabling novel applications.

### Splinted PNAmp duplicates target segments not bounded by direct repeats

Although PNAmp efficiently induces tandem duplication, its target segment must be bounded by direct repeats, severely limiting its applicability. We therefore sought to extend the targets of PNAmp to segments not bounded by direct repeats, but rather to arbitrary segments. A previous study caught our attention because it reported small fragment-driven DNA amplification (SFDA), which induces intrachromosomal tandem duplications using small DNA fragments with homology to two distant positions on the same chromosome.^52^ We hypothesized that PNAmp could be applied to any segment by providing such a small DNA fragment.

**Figure 6:**
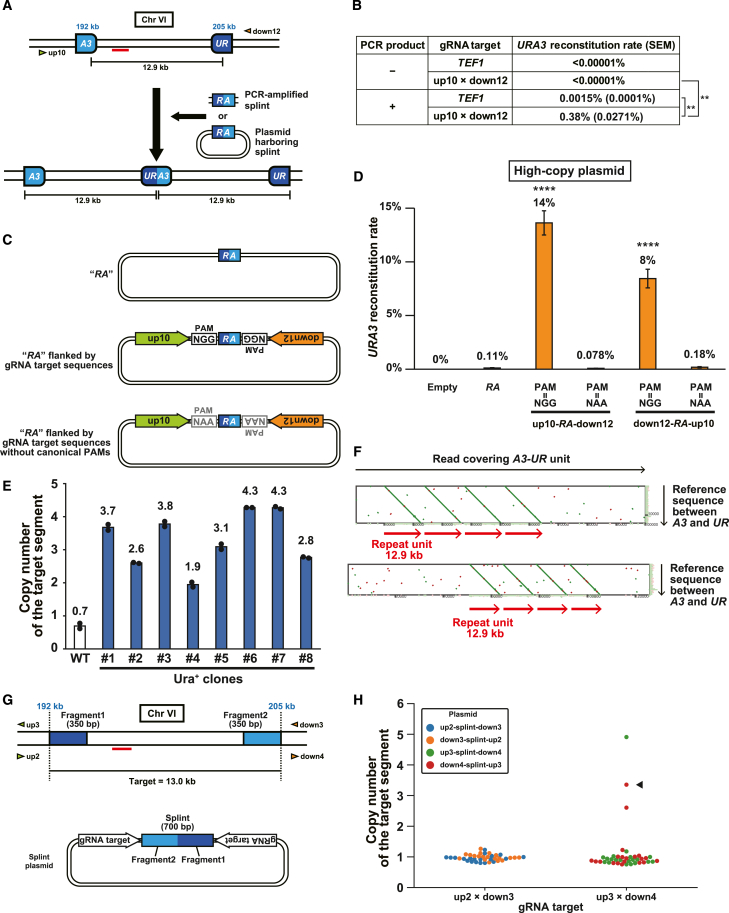
Splinted PNAmp duplicates target segments not bounded by direct repeats (A) Schematic of PNAmp with a splint DNA. Blue number, genomic coordinate; arrowhead, gRNA target; red bar, qPCR amplicon used in (E). (B) *URA3* reconstitution rates with splint PCR product transformation. Each experiment was performed 3 times. Statistical significance was examined using Student’s t test (∗∗*p* < 0.01). Without PCR product transformation, no Ura^+^ colonies appeared from >2 × 10^7^ colony-forming units (<0.00001%). (C) Schematic of splint plasmids. NGG, protospacer adjacent motif (PAM); NAA, mutated PAM. (D) *URA3* reconstitution rates of the strains carrying the indicated splint plasmids. Error bar, SEM (*n* = 3). ∗∗∗∗*p* < 0.0001 (Dunnett’s test). (E) Copy number of the target segment in 8 Ura^+^ clones determined by qPCR. These clones had the splint plasmid (down12-*RA*-up10, PAM = NGG) in (C). Dots indicate technical replicates for each clone. (F) Dot plots between nanopore reads of the Ura^+^ clone 6 in (E) and the reference sequence of the 12.9-kb segment. Of the 10,431 reads over 90 kb, 21 covered the entire amplicon. (G) Schematic of splinted PNAmp in a natural genomic context. Top, 13.0-kb target segment on chromosome VI, with 350-bp terminal portions incorporated in the 700-bp splint. Arrowhead, gRNA target; red bar, qPCR amplicon used in (H). Bottom, splint plasmid. (H) Copy-number distribution among clones randomly selected after PNAmp. Arrowhead, clone subjected to nanopore sequencing in Figure S7G.

To test this possibility, we constructed a new reporter system to detect *URA3* reconstitution using two fragments derived from the *URA3* gene, *A3* and *UR*, which are sequential in the original gene and have no overlapping sequence (Figure S7A). We integrated these fragments into the same positions on chromosome VI, described above (Figure 6A). We used PCR to generate a 400-bp “splint” fragment spanning the boundary of the *UR* and *A3* fragments, called *RA* (Figure 6A), transformed the new reporter strain without or with the PCR products, and induced the expression of nCas9 and gRNAs. Note that the splint transformation increased the *URA3* reconstitution rate >38,000-fold compared to the mock transformation (<0.00001% vs. 0.38%) (Figure 6B). Conversely, paired nicking resulted in a ∼250-fold increase in the efficiency of SFDA (0.0015% vs. 0.38%) (Figure 6B). The effect of splint transformation was also evident in cells harboring the reporter on chromosome IV, albeit with lower reconstitution rates (Figures S7B and S7C). These data collectively proved the principle of direct repeat-free PNAmp using a small splint DNA fragment. We thus called this method “splinted PNAmp.”

Although the splinted PNAmp eliminated the need for terminal direct repeats to delineate the target segments, its efficiency was much lower than the PNAmp of repeat-bounded target segments. To increase the efficiency of the splinted PNAmp, we tested providing the splint from the plasmid (Figures 6A and 6C, top). We first tried a low-copy, centromeric splint plasmid in the strain carrying the reporter on chromosome IV. However, the low-copy splint plasmid yielded an even lower efficiency than the PCR product (0.034% vs. 0.005%) (Figures S7C and S7D). We next used a high-copy, 2-μm splint plasmid because it should be present in the nucleus at a copy number of 40–60 per haploid cell,^53^ whereas the centromeric plasmid has a copy number of <10.^54^ With the high-copy splint plasmid, the reconstitution rate increased ∼6-fold to reach 0.03% (Figure S7D), underscoring the importance of copy number, but remained comparable to that with the PCR product (0.034%; Figure S7C). In the cells carrying the reporter on chromosome VI, the *URA3* reconstitution rate with the high-copy splint plasmid was 0.11% (Figure 6D), again, not exceeding that with the PCR product (0.38%; Figure 6B).

Notably, a previous study reported that the simultaneous nicking of donor plasmid and chromosomal target site induced homology-directed knockin more efficiently than simple nicking of the target site.^55^ We thus attempted to introduce nicks at the target-flanking sites on the genome and the splint-flanking sites on the plasmid. For this purpose, we inserted the gRNA target sequences into the plasmid to sandwich the splint (Figure 6C, center). In this setting, the gRNA pair induces two paired nicks, one on the genome and the other on the plasmid. With this two-paired nick method, the *URA3* reconstitution rate showed a remarkable increase to ∼15% in the chromosome VI reporter strain (Figure 6D), achieving an efficiency comparable to the PNAmp of repeat-bounded target segments. The qPCR results suggested duplication and quadruplication of the target segments (Figure 6E). Nanopore sequencing proved the quadruplication (Figure 6F). This method also improved the efficiency of the splinted PNAmp on chromosome IV (Figures S7D–S7F).

We then aimed to demonstrate splinted PNAmp in a natural genomic context. To achieve this, we targeted a 13-kb segment on chromosome VI and combined the two 350-bp sequences derived from both ends of this fragment to construct the splint fragment (Figure 6G, top). We designed the gRNA pair and splint plasmid so that the former, expressed from a Tet-on cassette integrated into the genome, ensures nCas9 to induce nicks that sandwich not only the genomic target segment but also the splint fragment on the latter (Figure 6G, bottom). Following induction of PNAmp, we quantified the target copy number in randomly selected clones using qPCR. While we did not detect duplication with one gRNA pair, we observed duplication/multiplication of the target segment in 3 out of 40 clones (7.5%) expressing the other gRNA pair (Figure 6H). Subsequent nanopore sequencing confirmed quadruplication of the target segment (Figure S7G).

Taken together, the splinted PNAmp with the two-paired nick method allows efficient duplication of genomic segments even when they lack terminal direct repeats, greatly expanding the targets of PNAmp.

### PNAmp is applicable to mammalian cells

**Figure 7:**
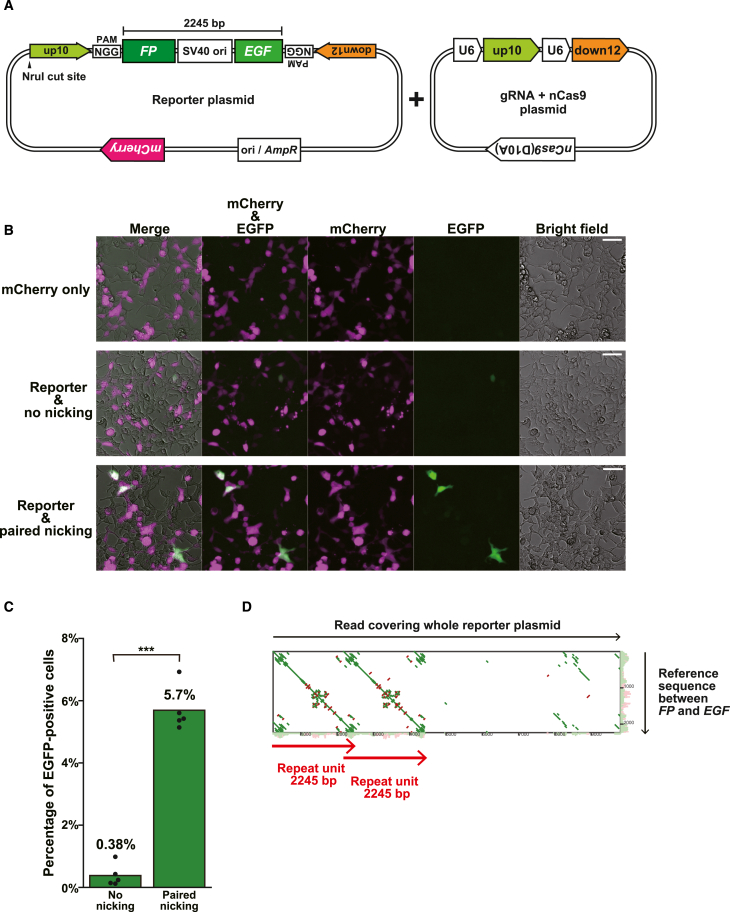
PNAmp is applicable to mammalian cells (A) Schematic of PNAmp in HEK293T cells. Left, duplication reporter plasmid. Right, co-expression plasmid for gRNAs and nCas9. U6, human U6 small nuclear RNA promoter. (B) Microscopic images of transfected cells. Top row, cells co-transfected with a reporter plasmid derivative lacking *FP* and *EGF* and a gRNA + nCas9 plasmid encoding no gRNA. Center row, cells co-transfected with the reporter plasmid and the gRNA + nCas9 plasmid encoding no gRNA. Bottom row, cells co-transfected with the reporter plasmid and the gRNA + nCas9 plasmid expressing nCas9 targeting up10 and down12. Scale bar, 50 μm. (C) Frequency of EGFP^+^ cells among mCherry^+^ cells. Statistical significance was examined using Student’s t test (∗∗∗*p* < 0.001). (D) Dot plot between a representative nanopore read and the reference sequence of the 2,245-bp target segment bounded by *FP* and *EGF*. Of the 33,267 plasmid reads, 116 supported the intended duplication.

Finally, we investigated the feasibility of PNAmp in mammalian cells, which are known to have lower homologous recombination efficiencies than budding yeast. For this purpose, we conducted experiments using an episomal duplication reporter system (Figure 7A). The reporter plasmid contains two fragments, *FP* and *EGF*, derived from the *EGFP* gene. These fragments share a 350-bp sequence designated *F* and are flanked by gRNA target sequences, up10 and down12, derived from yeast chromosome IV (Figure 7A, left). Crucially, the reporter plasmid also contains the replication origin of Simian Virus 40 (SV40 ori) between *FP* and *EGF* to fulfill the configurational requirement for PNAmp (Figure 7A, left). Additionally, it encodes the *mCherry* gene as a transfection marker (Figure 7A, left). We co-transfected the reporter plasmid with a plasmid co-expressing nCas9 and gRNAs (nCas9 + gRNA plasmid) (Figure 7A, right) into HEK293T cells expressing the large T antigen that activates SV40 ori and assessed EGFP fluorescence to estimate PNAmp efficiency. Cells transfected with the negative control nCas9 + gRNA plasmid lacking any gRNA barely exhibited EGFP signals, and even if they did, the signal was rather weak (Figure 7B). Conversely, a subset of cells transfected with the nCas9 + gRNA plasmid encoding the gRNA pair targeting up10 and down12 showed intense EGFP signals (Figure 7B). Quantitative microscopy revealed that paired nicking significantly increased the frequency of EGFP^+^ cells among mCherry^+^ cells (Figures 7C and S7H). Importantly, a reporter plasmid derivative lacking the SV40 ori did not result in an increase in EGFP^+^ cells (Figure S7H). Nanopore sequencing of plasmid DNAs recovered from EGFP^+^ cells confirmed the intended duplication (Figure 7D). Taken together, these findings demonstrate that the duplication leading to *EGFP* reconstitution depends on both paired nicking and DNA replication, providing evidence for PNAmp in mammalian cells.

## Discussion

In this study, we utilized budding yeast to establish PNAmp as an efficient method for inducing targeted duplication of even segments as large as ∼1 Mb. We also presented evidence supporting PNAmp in mammalian cells.

The SSA model effectively explains the canonical PNAmp with *trans*-nicking but not the non-canonical PNAmp with *cis*-nicking, where two one-ended DSBs occur on the same chromatid to excise the target segment. The excised segment can be circularized via SSA between the terminal repeats to integrate into the intact chromatid through homologous recombination, resulting in the target duplication. Because the non-canonical PNAmp leaves the unnicked strand intact, it potentially serves as a unique method to induce tandem SDs, although it lags behind the canonical PNAmp in inducing large SDs (unpublished data). While the mechanism described above needs to be validated in future studies, its similarity to the mechanism proposed for *de novo* deletions and duplications at recombination hotspots in mouse germ lines^56^ suggests a potential application of the non-canonical PNAmp in generating non-tandem SDs, provided that ectopic integration of excised DNA can be enhanced.

Critical determinants of PNAmp include gRNA performance, which varies substantially, likely influenced by complex factors. When used individually, gRNAs targeting up11 and down11 showed the highest (1.9%) and second highest (0.7%) efficiencies, respectively, whereas others showed low efficiencies ranging from 0.1% to 0.3% (Figure 1). However, when used in pairs, a combination of low-efficiency gRNAs targeting up12 and down12 (up12 × down12) showed the highest efficiency (35%), comparable to that of a combination of high-efficiency gRNAs (up11 × down11) (34%) (Figure S2). Intriguingly, up11 × down11 outperformed and underperformed up10 × down12 in PNAmp on chromosomes IV and VI, respectively (Figures S2 and 4B). Furthermore, the performance seemed independent of whether the gRNA targets are on the leading or lagging strand templates: the two best gRNA pairs, up11 × down11 and up12 × down12, guide nCas9 to nick the leading and lagging strand templates, respectively (Figure S2). These results collectively indicate that we cannot predict the performance of gRNA pairs and underscore the need for systematic evaluation.

Replication initiation from within the target segment is another critical factor for PNAmp. However, we observed that PNAmp occurs at a lower frequency even in a strain lacking annotated ARSs in the target segment, suggesting involvement of non-canonical, distributive replication initiation detected by Pu-seq (Figure 3). While we cannot predict non-canonical origins, these results indicate a possibility of expanding PNAmp to segments apparently devoid of replication origins. PNAmp critically depends on the timing of fork arrival and nick repair. Considerations of non-ideal conditions for PNAmp, such as nick closure before fork arrival, prompted us to propose alternative models that could explain observations challenging the standard model (Figure S4). The contribution of these alternative mechanisms to PNAmp remains elusive and warrants further investigation.

Terminal repeats of target segments are also crucial for PNAmp. However, such identical sequences are rarely found in the actual genomic context. Even transposable elements often show significant sequence divergence. Nevertheless, PNAmp can utilize naturally occurring homologous but non-identical sequences, especially when MMR is compromised (Figure S5). These findings not only help to expand the applicability of PNAmp to natural sequence contexts but also suggest the potential involvement of PNAmp-like events in evolutionary processes. Moreover, we demonstrated that short splint DNA allows PNAmp to operate without identical or homologous sequences at target segment ends (Figure 6). Further optimization of splinted PNAmp will fully exploit its potential to design SDs with minimal constraints.

### Limitations of the study

PNAmp is replication coupled and applicable only to dividing cells. Its performance depends on nick availability at replication fork arrival, which is determined by gRNAs and replication status around the target segment, making it currently unpredictable. Although we have presented evidence for mammalian PNAmp, further studies are needed to determine its efficiency when utilizing broad replication initiation zones in the mammalian genome.

## STAR★Methods

### Key resources table

**Table:** 

REAGENT or RESOURCE	SOURCE	IDENTIFIER
**Antibodies**		

Monoclonal ANTI-FLAG® M2 antibody produced in mouse, clone M2, purified immunoglobulin (Purified IgG1 subclass)	Sigma-Aldrich	Cat# F3165; RRID:AB_259529
Goat Anti-Mouse IgG H&L (HRP)	Abcam	Cat# ab6789; RRID: AB_955439
Alpha Tubulin antibody (YOL1/34)	GeneTex	Cat# GTX26161; RRID: AB_385177
Goat Anti-Rat IgG H&L (HRP)	Abcam	Cat# ab97057; RRID:AB_10680316

**Bacterial and virus strains**		

DH5α high Champion™ cell	SMOBIO	Cat# CC5202

**Chemicals, peptides, and recombinant proteins**		

D-(+)-Raffinose Pentahydrate	Wako	Cat# 17629-30-0
Doxycycline hydrochloride	Apollo scientific	Cat# BID0121

**Critical commercial assays**		

KOD One® PCR Master Mix (Dye-free 2×PCR Master Mix)	TOYOBO	Cat# KMM-101
KOD SYBR® qPCR Mix	TOYOBO	Cat# QKD-201
Chelex 100 Chelating Resin, biotechnology grade, 100–200 mesh, sodium form	Bio-Rad	Cat# 1432832
Quick-DNA Fungal/Bacterial Miniprep Kit	ZYMO RESEARCH	Cat# D6005
Monarch HMW DNA Extraction Kit for Tissue	NEB	Cat# T3060L
QIAGEN Genomic-Tip 100/G	QIAGEN	Cat# 10243
EndoFree Plasmid Maxi Kit	QIAGEN	Cat# 12362
NEB Golden Gate Assembly Kit (BsaI-HF v2)	NEB	Cat# E1601L
NEBuilder HiFi DNA Assembly Master Mix	NEB	Cat# E2621L
CHEF Genomic DNA Plug Kits	Bio-Rad	Cat# 1703491
7.5% Mini-PROTEAN® TGX™ Precast Protein Gels, 12-wells	Bio-Rad	Cat# 4561025
Trans-Blot Turbo Mini 0.2μm PVDF Transfer Packs	Bio-Rad	Cat# 1704156
iBind™ Solution Kit	Thermo Fisher	Cat# SLF1020
iBind™ Cards	Thermo Fisher	Cat# SLF1010
Clarity Max Western ECL Substrate	Bio-Rad	Cat# 1705062
AlkPhos Direct Labeling Module for 25 labellings	Cytiva	Cat# RPN3680
CDP-Star Detection Reagent for 2,500 cm^2^ membrane	Cytiva	Cat# RPN3682
AlkPhos Direct Hybridization Buffer for 5,000 cmˆ2 membrane	Cytiva	Cat# RPN3688
Lipofectamine 3000® Reagent	Thermo Fisher	Cat# L3000008
Quick-DNA Microprep Kit	ZYMO RESEARCH	Cat# D3020
Ligation Sequencing Kit	Oxford Nanopore Technologies	SQK-LSK109
Native Barcoding Kit	Oxford Nanopore Technologies	EXP-NBD104
Native Barcoding Kit 96 V14	Oxford Nanopore Technologies	SQK-NBD114.96
MinION Flow Cell (R9.4.1)	Oxford Nanopore Technologies	FLO-MIN106D
MinION Flow Cell (R10.4.1)	Oxford Nanopore Technologies	FLO-MIN114
Flongle Flow Cell (R10.4.1)	Oxford Nanopore Technologies	FLO-FLG114

**Deposited data**		

*S. cerevisiae* S288C reference genome: sacCer3	Saccharomyces Genome Database	https://www.ncbi.nlm.nih.gov/datasets/genome/GCF_000146045.2/
Raw sequence data	This paper	DDBJ BioProject database PRJDB16187

**Experimental models: Cell lines**		

HEK293T	RIKEN BRC	RBRC-RCB2202

**Experimental models: Organisms/strains**		

*S*. *cerevisiae*: Strain background: BY4741		
All other synthetic yeast strains used in this paper, listed in Table S1	This paper	N/A

**Oligonucleotides**		

All oligonucleotides used in this paper, listed in Table S3	This paper	N/A

**Recombinant DNA**		

pTopo pLox hPGK-Puro pA pLox	Penev et al.^73^	Addgene plasmid # 171048
pKLV-EF1amCherry-W	Au et al.^74^	Addgene plasmid #159295
pcDNA3-EGFP	Doug Golenbock	Addgene plasmid # 13031
AIO-Puro	Chiang et al.^75^	
All other plasmids used in this paper, listed in Table S2	This paper	N/A

**Software and algorithms**		

MinKNOW	Oxford Nanopore Technologies	https://community.nanoporetech.com/downloads?from=support
Guppy v4.0.14	Oxford Nanopore Technologies	https://community.nanoporetech.com/downloads?from=support
Minimap2 v2.17-r941	Li^64^	https://github.com/lh3/minimap2
samtools v1.10	Danecek et al.^65^	https://github.com/samtools/samtools
bedtools v2.27.1	Quinlan and Hall^66^	https://github.com/arq5x/bedtools2
Bedgraph_norm_ratio.py	Satoshi Okada	https://doi.org/10.5281/zenodo.11515696
minialign	Hajime Suzuki	https://github.com/ocxtal/minialign
seqkit v0.15.0	Shen et al.^68^	https://github.com/shenwei356/seqkit
YASS	Noe and Kucherov^67^	https://bioinfo.univ-lille.fr/yass/index.php
Bowtie2 v2.3.5	Langmead et al.^72^	https://github.com/BenLangmead/bowtie2
sam-dup-align-exclude-v2.pl	Yasukazu Daigaku	https://doi.org/10.5281/zenodo.11541288
pe-sam-to-bincount.pl	Yasukazu Daigaku	https://doi.org/10.5281/zenodo.11541286
bincount-csv_to_pol-usage-wig.R	Yasukazu Daigaku	https://doi.org/10.5281/zenodo.7273730
pol-usage-wig_to_ini-index-wig.R	Yasukazu Daigaku	https://doi.org/10.5281/zenodo.7273730
blast	Altschul et al.^76^	N/A

### Resource availability

#### Lead contact

Further information and requests for resources and reagents should be directed to and will be fulfilled by the lead contact, Takashi Ito (ito.takashi.352@m.kyushu-u.ac.jp).

#### Materials availability

Requests for the generated plasmids and strains in this study should be directed to the lead contact, Takashi Ito (ito.takashi.352@m.kyushu-u.ac.jp).

#### Data and code availability

• All raw sequencing data used in this study were deposited with links to BioProject accession number PRJDB16187 in the DDBJ BioProject database.
• All original codes used in this study are available at Zenodo, including: 1) Bedgraph_norm_ratio.py for calculating normalized nanopore read counts (https://doi.org/10.5281/zenodo.11515696), 2) sam-dup-align-exclude-v2.pl for excluding multiply mapped Pu-seq reads (https://doi.org/10.5281/zenodo.11541288), 3) pe-sam-to-bincount.pl for calculating Pu-seq read counts in 100 bp bins (https://doi.org/10.5281/zenodo.11541286), 4) bincount-csv_to_pol-usage-wig.R for calculating the polymerase usage (https://doi.org/10.5281/zenodo.7273730), and 5) pol-usage-wig_to_ini-index-wig.R for calculating the initiation index (https://doi.org/10.5281/zenodo.7273730).

### Experimental model and subject details

The budding yeast *Saccharomyces cerevisiae* was used as the primary experimental model in the study. The haploid yeast strain BY4741 was used as the parental strain. As a model of mammalian cells, the human female embryonic kidney-derived cell line HEK293T was purchased from RIKEN BRC (catalog number RBRC-RCB2202) and cultured in Dulbecco’s modified Eagle medium (DMEM) (Gibco, catalog number 11885084) at 37°C.

### Method details

#### Yeast strains

Yeast strains used in this study are listed in Table S1. All yeast strains used in this study are derived from BY4741.^57^ Standard culture media and genetic methods were used in this study.^58^ We deleted a gene of interest by transforming a *NatMX* cassette flanked by the upstream and downstream sequences of the open reading frame (ORF) of the gene to be disrupted,^59^ which was amplified from a relevant plasmid (Table S2) by PCR using appropriate primers (Table S3). The genomic coordinates on chromosome IV in the PNAmp reporter strains are shifted by ∼10 kb from those in the SacCer3 reference genome because they carry the nCas9 expression cassette at the *HO* locus on chromosome IV. In addition, the insertion of reporter fragments altered the genomic coordinates on chromosome IV or VI. These differences are summarized for the strains used in Pu-seq (Table S4) and nanopore sequencing (Tables S5 and S6).

#### Yeast genome editing

We performed genome editing for the insertion of *RA3* and *UR* fragments, the insertion of *ARS305*, the deletion of *ARS418* and *ARS419*, and the amino acid substitution of *POL2* and *POL3* for Pu-seq. Genome editing was performed as described previousy^60^ with some modifications. All genome editing plasmids and primers used in this study are listed in the Tables S2 and S3. Each genome editing plasmid encodes SpCas9 or enAsCas12a fused to SV40 nuclear localization signal and gRNA, both under the control of the *GAL1* promoter. To design the gRNAs, we used CRISPOR to select target sequences.^61^ PCR-generated donor fragments containing flanking sequences of cleavage sites are used for insertion or deletion. We transformed the genome editing plasmids and donor fragments into the host cells, which were spread on agar plates containing YPA medium supplemented with 2% galactose and 200 μg/mL G418 (Nacalai tesque and InvivoGen) for selection. After incubation at 30°C for 3 days, colonies were picked and streaked onto new plates. To confirm the successful genome editing, we performed PCR to check the sequence length covering the edited region. We verified the sequence of the PCR product of the edited region by Sanger sequencing. After performing genome editing, the cells were cultured in YPD liquid medium to drop the genome editing plasmids, and isolated colonies were streaked on YPD and YPD + G418 medium, and we picked up and preserved the G418-sensitive clones for further experiments.

#### Yeast plasmids

All plasmids used in this study are listed in Table S2. All primers used for plasmid construction were purchased from Eurofins Genomics and Sigma-Aldrich Japan. Plasmids were constructed using HiFi DNA Assembly or Golden Gate Assembly (New England Biolabs) and transformed into DH5α high Champion cells.

For gene disruption, plasmids were used as the templates for PCR to prepare the *NatMX* cassette flanked by the upstream and downstream sequences of the ORF of the gene of interest. These template plasmids were constructed by inserting the upstream and the downstream sequences of the ORF into the *NatMX* on a *YCp-NatMX* plasmid, which has restriction enzyme recognition sites at the upstream and downstream of the *NatMX* cassette.

The centromeric plasmids for the induction of nicks carry genes for one or two gRNAs, all under the control of the Tet-On system,^62^ in which these genes are expressed by the presence of a reverse tetracycline-controlled transcription factor (rtTA) and a tetracycline derivative, doxycycline (Dox). The gene encoding the rtTA under the control of the constitutive CMV promoter is also encoded on these plasmids.

The plasmids carrying the fragments derived from a region on chromosome IV, where the initiation index peaks were observed, were used for the replication activity assays. These plasmids were derived from *YCp-URA3-ARS305*, a centromeric plasmid harboring *URA3* and *ARS305* flanked by restriction enzyme recognition sites. *ARS305* on this plasmid was replaced with the fragments derived from chromosome IV (for details of the fragments, see "Replication activity assay" section) or *ARS604,* or deleted to construct the empty negative control plasmid.

For the construction of the plasmids encoding the "splint" DNA for the *URA3* reconstitution, *YCp-KanMX* or *YEp-KanMX* were used as the backbone, which was inserted with a 400-bp splint, *RA*, covering the 200-bp region from the 3′-end of the *UR* fragment and the 200-bp region from the 5′-end of *A3* fragment. The splint sequence is flanked by gRNA target sequences accompanied by canonical PAM (NGG) or unfunctional PAM (NAA). Similarly, the plasmid bearing a 700-bp splint DNA for a genomic segment on chromosome VI was constructed.

#### Quantitative PCR (qPCR)

Genomic DNA for qPCR was extracted with "GCpreps method," in which cells were lysed by vortex mixing with glass beads and boiled with a metal chelating resin.^63^ From 100 μL of cell suspension of each sample, each qPCR solution contained 1 μL of extracted genomic DNA and 4 pmol of each forward and reverse primer, 10 μL of KOD SYBR qPCR Mix (TOYOBO) containing 0.08 μL of 50 × ROX reference dye. Each qPCR was performed in technically duplicates using QuantStudio3 (Applied Biosystems). The amplification condition was an initial denaturation at 95°C for 30 s followed by 40 times of a 3-step thermal cycle consisting of 95°C for 10 s, 58°C for 30 s, and 68°C for 10 s. All qPCR runs included 10-fold serial dilutions to generate standard curves. The amount of target sequences was indicated as the average value of technical duplicates and normalized to that of *ACT1*.

#### Whole-genome nanopore sequencing

To confirm the SD induced by PNAmp, we performed whole-genome sequencing using the Oxford Nanopore sequencer. Genomic DNA was extracted using Quick-DNA Fungal/Bacterial Miniprep Kit (ZYMO RESEARCH) to assess read coverage of the target segment (Figures 1E, 4B, and S5B) or Monarch HMW DNA Extraction Kit for Tissue (New England Biolabs) to obtain the long reads covering the target segment (Figures 1F, 5E, 6F, S5C, S7F, and S7G) according to manufacturer’s instructions. DNA libraries were prepared using the ligation kit SQK-LSK109 or SQK-LSK114 (Oxford Nanopore Technologies) with or without the barcoding kit EXP-NBD104 (Oxford Nanopore Technologies), or solely with SQK-NBD114.96 (Oxford Nanopore Technologies). The prepared libraries were sequenced using the FLO-MIN106D R9.4.1 or FLO-MIN114 R10.4.1 flowcell and the MinION sequencer (Oxford Nanopore Technologies). The sequencer was controlled by the MinKNOW operating software. The run time was 72 h. Base calling was performed with Guppy v4.0.14 or Dorado v0.4.3. The obtained reads were mapped using minimap2^64^ to the customized SacCer3 reference genome sequence, where Ty elements, ribosomal DNA, *CUP1* array, and *ENA1/2/5* array were masked as “N”. Generated sam files were binarized using samtools^65^ and further transformed into bedgraph using bedtools.^66^ To assess read coverage, normalized read counts were calculated by dividing the read counts for individual nucleotides in the target segment by the average read count of the entire genome using a python script (https://doi.org/10.5281/zenodo.11515696). Average normalized read counts in Figures S5D and S5E were calculated by dividing the sum of normalized read counts for all nucleotides in the target region by the total number of nucleotides in that segment.

#### Dot plot analysis

We generated dot plots using YASS.^67^ We first filtered out the short reads using seqkit.^68^ From the filtered reads, we next identified those covering the duplicated segments using 1-kb sequences upstream of *RA3* and downstream of *UR* as queries in minialign (https://github.com/ocxtal/minialign) and used them as the first input sequence for YASS. As a second input, we used the reference sequences of the target segment flanked by *RA3* and *UR* on chromosome IV or VI. To search for reads covering the duplication junction in the PNAmp of the Ty1-bounded region, we used two 1-kb sequences, one upstream of *YDRWTy1-5* and the other downstream of *YDRWTy1-4*, as queries.

#### Induction of PNAmp

We first constructed a strain harboring the genetic reporter system to detect the SD. We obtained the two fragments derived from the *URA3* gene, *RA3* and *UR*, which have a 391-bp overlapping sequence, by PCR using the primers and the YCplac33 plasmid as the template (listed in Tables S2 and S3). Then, we inserted these fragments into 555 kb and 602 kb positions on chromosome IV, respectively, by genome editing (Note that the inserted positions of *RA3* and *UR* are indicated in genomic coordinates in chromosome IV of the PNAmp strains carrying the nCas9 expressing cassette in the *HO* locus in chromosome IV. In this genomic coordinate, the sequence downstream of the *HO* is shifted by ∼10 kb compared to the standard reference sequence of the S288C strain). Next, we transformed the reporter strains with plasmids carrying the gRNAs expression cassette by Tet-on system. To induce PNAmp, we first streaked the frozen stock of the cells on agar plates containing SC medium supplemented with 0.1% monosodium glutamate (MSG, Nacalai tesque), 200 μg/mL G418 (Nacalai tesque and InvivoGen), and 0.1% 5-Fluoroorotic acid (5-FOA) (Apollo Scientific), which eliminates cells with spontaneously reconstituted *URA3* gene, and then inoculated the cells into 2 mL YPD + G418 liquid medium for subsequent growth at 30°C for 14 h. Next, we inoculated 40 μL of the cultured cells into 2 mL YPD + G418 and incubated the culture at 30°C for 5–6 h to obtain the cells in the logarithmic phase. Then, we further inoculated the cells into 10 mL YPRaffinose (2%) supplemented with 200 μg/mL G418 and 10 μg/mL Dox to express nCas9 and gRNAs and grew the cells for 14 h until they reached to OD_600_ of ∼0.4. Finally, we spread 50–300 colony-forming units (CFUs) on SC agar plates and 10-times more CFUs on SC−Ura agar plates. After incubating the plates at 30°C for 3 days, we counted the colonies on both plates and calculated the *URA3* reconstitution rate using the following formula, [the number of the colonies on the SC−Ura plate]/10 × [the number of the colonies on the SC plate].

To construct a series of strains carrying the genetic reporter system on chromosome IV with different distances between *RA3* and *UR*, we first deleted the region covering *UR* and the downstream gRNA target sequences from the strain carrying *RA3* in 555 kb and *UR* in 602 kb on chromosome IV by genome-editing. We then inserted *UR* and the gRNA target sequences into different positions by genome editing. The inserted fragment harboring *UR* and the downstream gRNA target sequences was prepared by PCR using the genomic DNA of the strain harboring *UR* in 602 kb on chromosome IV as a template. To induce PNAmp in these strains, gRNAs targeting up11 and down11 were used. The culture conditions to induce PNAmp in these strains were the same as those described above.

To construct the strain carrying the genetic reporter system on chromosome VI, we further deleted *RA3* and the upstream gRNA target sequences on chromosome IV of the strain encoding only *RA3* in 555 kb on chromosome IV, in which the region covering *UR* and the downstream gRNA target sequences were deleted (described above). By this genome editing, we obtained a strain lacking *RA3* and the upstream gRNA target sequence and *UR* and the downstream gRNA target sequence. For this strain, we sequentially inserted *RA3* and the upstream gRNA target sequence into 192 kb on chromosome VI, and *UR* and the downstream gRNA target sequence into 205 kb on chromosome VI. The culture conditions to induce PNAmp of this strain were the same as the other strains described above.

To construct the strain carrying a fluorescent protein gene in the genetic reporter on chromosome VI, we used genome editing to integrate an mNeonGreen expression cassette flanked by a hygromycin B resistance gene (*HphMX*, for selecting successfully inserted clones) into the position upstream of *ARS607*. Cells were selected on a medium supplemented with 300 mg/L of Hygromycin B (Nacalai tesque). After PNAmp induction, we grew the isolated clones and measured the intensity of mNeonGreen fluorescence using the EVOS M7000 Imaging system (Thermo Fisher Scientific). Genomic DNA was then extracted from these incubated colonies and the copy number of the target segment was measured by qPCR.

To induce PNAmp at the region bounded by Ty1 elements or LTRs, gRNAs were designed upstream of *YDRWTy1-4* (Ty_up4), downstream of *YDRWTy1-5* (Ty_down1), upstream of *YDRWdelta25* (LTR_up4) and downstream of *YDRWdelta26* (LTR_down2). Ty_up4 and Ty_down1, LTR_up4 and LTR_down2 were paired and encoded on the plasmid to be expressed by the Tet-on system as described above. After inducing PNAmp in the cells harboring one of these two plasmids, the cells were plated on the SC media. The colonies on the media were pooled, and the genomic DNA was extracted for whole genome sequencing.

#### Splinted PNAmp

We first constructed a strain in which chromosome IV carried two fragments derived from the *URA3* gene, *A3* and *UR*, without overlapping sequence, at a distance of 47 kb. Each fragment was inserted by genome editing as described above, but the fragments were obtained by PCR using different primers (Table S3).

For PNAmp with a PCR product as a splint, we prepared a 400-bp PCR product spanning the boundary of *UR* and *A3*, using YCplac33 as a template. The frozen stocks of the strains harboring *A3* and *UR* on chromosome IV, which carries the plasmid to express gRNA(s) targeting up11 and down11, or *TEF1* on chromosome XVI as a negative control, were streaked on the SC agar plates supplemented with 0.1% MSG, 200 μg/mL G418, and 0.1% 5-FOA. These strains were inoculated into YPD + G418 liquid medium and cultured at 30°C for 14 h. We transformed 30 pmol of the 400-bp PCR product into 0.08 OD_600_ units of cultured cells, inoculated the transformants into 10 mL YPRaffinose liquid medium supplemented with 200 μg/mL G418 and 10 μg/mL Dox, and grew the cells at 30°C for 15 h. These cultured cells were plated on SC or SC−Ura agar plates and incubated at 30°C for 3 days, the number of the colonies was counted, and the *URA3* reconstitution rates were calculated as described above.

For PNAmp with the plasmids harboring the splint sequence, we introduced the gRNAs expression cassette into a safe harbor locus on chromosome X^69^ by plasmid integration. Into these strains, we further transformed the centromeric or 2-micron type plasmids harboring the 400-bp splint sequence, with or without being flanked by gRNA target sequences, whose PAMs were canonical or unfunctional. We also transformed the empty plasmids lacking the splint sequence. The procedure to induce PNAmp in these strains was the same as in the experiments of PNAmp with PCR products as a splint.

For splinted PNAmp in a natural genomic context, we introduced the gRNAs expression cassette into chromosome X as described above to generate two strains, one expressing gRNAs targeting up2 and down3 and the other expressing gRNAs targeting up3 and down4. We further transformed these strains with the 2-micron type plasmid harboring the 700-bp splint sequence generated by fusing the two 350-bp fragments derived from both ends of the target segment. This 700-bp splint sequence was sandwiched by the gRNA target sequences in two arrangement patterns (upX-splint-downY or downY-splint-upX). Accordingly, we prepared four splint plasmids in total, each coding one of the four construct: up2-splint-down3, down3-splint-up2, up3-splint-down4, or down4-splint-up3. The plasmids coding up2-splint-down3 or down3-splint-up2 were transformed into the strain expressing gRNAs targeting up2 and down3, and the plasmids coding up3-splint-down4 or down4-splint-up3 were transformed into the strain expressing gRNAs targeting up3 and down4. After PNAmp induction, these four strains were plated on the SC media, and 20 clones of each strain were tested for copy number measurements.

#### Western blotting

The expression of FLAG-tagged Rad52 was analyzed by western blotting. Proteins were extracted from 1 × 10^7^ cells as described previously.^70^ Proteins were separated with 7.5% sodium dodecyl sulfate-polyacrylamide gel electrophoresis using 7.5% Mini-PROTEAN TGX Precast Gel (Bio-Rad). Transfer to the membrane was performed with Trans-Blot Turbo system according to the manufacturer’s protocol. Antibody reactions were performed using iBind Western System (Thermo Fisher Scientific) according to the manufacturer’s protocol. Primary and secondary antibodies to detect Rad52-FLAG were FLAG M2 mouse monoclonal antibody (1:1000, Sigma-Aldrich) and Goat anti-mouse IgG H&L HRP (1:2000, Abcam, ab6789), respectively. Primary and secondary antibodies to detect α-tubulin (as loading control) were anti-alpha Tubulin antibody [YOL1/34] (1:2000, GeneTex) and goat Anti-Rat IgG H&L (HRP) (1:2000, Abcam), respectively. Following incubation with Clarity Max Western ECL Substrate (Bio-Rad), chemiluminescent signals were detected with ChemiDoc Touch system (Bio-Rad). Gel images were processed with ImageJ software. The process involved cropping and altering window-level settings.

#### Polymerase-usage sequencing (Pu-seq)

We employed polymerase-usage sequencing (Pu-seq) to reveal the status of replication initiation around the target segment of PNAmp.^71^ For the Pu-seq, we used genome editing to construct strains carrying *pol2-M644G*^46^ and *pol3-L612G*,^47^ encoding the mutant DNA polymerases ε (Polε) and δ (Polδ), respectively, in the background of *rnh201*Δ. In these strains, ribonucleotides are stably incorporated into the strand that the respective polymerases synthesize, leading strand and lagging strand. As a control, we also deleted *RNH201* in the strain encoding the wild-type DNA polymerases. We constructed these mutants in both backgrounds of *ARS418 ARS419* and *ars418*Δ *ars419*Δ, resulting in a total of 6 strains. We next cultured these strains in 100 mL of YPD medium at 30°C for 14 h, then harvested 7 × 10^9^ cells, and extracted genomic DNA using QIAGEN Genomic-tip 100/G (QIAGEN), according to the manufacturer’s instructions. For library preparation, 20 μg of genomic DNA was treated with 300 mM NaOH at 55°C for 2 h, then loaded onto a 1.5% agarose gel, and run at 100 V for 100 min. The gel was stained with acridine orange for 2 h. Fragments of 300–2,000 bp were excised from the gel and isolated using NucleoSpin Gel and PCR Clean-up (Macherey-Nagel). Library preparation was performed as previously described.^71^ The libraries were sequenced on the Illumina Hiseq X platform.

For each sample, at least 32 million pair-end reads were generated. Using Bowtie2 (version 2.3.5),^72^ raw reads were aligned to the customized SacCer3 reference genome (Table S4). We masked Ty elements, ribosomal DNA, *CUP1* array, and *ENA1/2/5* array with “N”. The reads aligned to multiple genomic locations with the same mismatch scores (AS and XS scores as outputted by Bowtie2) were excluded using a custom Perl script: sam-dup-align-exclude-v2.pl (https://doi.org/10.5281/zenodo.11541288). The position of the 5′ end of each R1 read (corresponding to the 5′ end of ssDNA hydrolyzed by alkaline treatment) was determined, and the number of reads in 100 bp bins across the genome was counted separately for the Watson and Crick strands using a custom Perl script: pe-sam-to-bincount.pl (https://doi.org/10.5281/zenodo.11541286). This generated the four datasets in separate csv files for the analysis of each polymerase described below.

In the case of Polε: at the chromosome coordinate x, N_w_^ε^(x) is the count for *pol2-M644G rnh201*Δ on the Watson strand; N_c_^ε^(x) is the count for *pol2-M644G rnh201*Δ on the Crick strand; N_w_^+^(x) is the count for POL^+^ *rnh201*Δ on the Watson strand; N_c_^+^(x) is the count for POL^+^ *rnh201*Δ on the Crick strand. The datasets were normalized using the total number of reads: e.g., N’wε (x) = N_w_^ε^(x)/Σ N_w_^ε^ for the Polε mutant on the Watson strand. These normalized genomic bin data of the Polε mutant were divided by those of the control strain to calculate relative polymerase usage: e.g., E_w_(x) = N’wε (x)/N’w+ (x) for Polε usage on the Watson strand; E_c_(x) = N’cε (x)/N’c+ (x) for Polε usage on the Crick strand. The equivalent analysis was performed to obtain the usage of Polδ on both strands: D_w_(x) and D_c_(x). When these data were plotted, they were smoothed using a moving average of 2m + 1, where m is 3 in this study. Thus, the data point for each bin is an average of 2m + 1 bins: the point of origin and the m bins on either side. This analysis was performed using a custom R-script: bincount-csv_to_pol-usage-wig.R (https://doi.org/10.5281/zenodo.7273730). In Figure S3A, we plotted E_w_(x) and D_w_(x), or E_c_(x) and D_c_(x), according to the chromosome coordinate, smoothed using the value m = 1. In Figures 3D and 3E, we further normalized the polymerase usage as, E_w_(x)/[E_w_(x) + D_w_(x)] and D_w_(x)/[E_w_(x) + D_w_(x)] and plotted according to the chromosome coordinate. The equivalent calculation was performed to E_c_(x) and D_c_(x) and plotted.

Initiation index was calculated as follows. The difference between each neighboring date point of polymerase usage was calculated as ΔE_w_(x), ΔE_c_(x), ΔD_w_(x), and ΔD_c_(x), with E_w_(x), E_c_(x), D_w_(x), and D_c_(x), which were smoothed using the value of m = 3. These differential data were further smoothed, using the value m = 3. At any position where all four polymerase profiles exhibit consistent patterns for the initiation of bidirectional replication forks (ΔE_w_(x) > 0 ∩ ΔE_c_(x) < 0 ∩ ΔD_w_(x) < 0 ∩ ΔD_c_(x) > 0), or patterns consistent with the merging of two forks (ΔE_w_(x) < 0 ∩ ΔE_c_(x) > 0 ∩ ΔD_w_(x) > 0 ∩ ΔD_c_(x) < 0), an initiation index was defined as: Ini(x) = ΔEw(x) − ΔEc(x) − ΔDw(x)+ ΔDc(x). These data were subjected to *Z* score normalization (mean = 0, standard deviation = 1) and Z(0) were subtracted to maintain the original + or − information, representing increased levels of replication initiation and termination in the cell population, respectively. This analysis was performed using a custom R-script: pol-usage-wig_to_ini-index-wig.R (https://doi.org/10.5281/zenodo.7273730).

Replication fork directionality (RFD) was calculated by subtracting the polymerase profiles of leftward moving fork signals from rightward moving fork signals. We calculated RFD as (E_w_(x) − E_c_(x) − ΔD_w_(x) + ΔD_c_(x))/(E_w_(x) + E_c_(x) + ΔD_w_(x) + ΔD_c_(x)).

#### Replication activity assay

To investigate the replication activity of the region indicated by the Pu-seq result of the *ars418*Δ *ars419*Δ strain (Figure 3E), we divided the region into five 1-kb fragments, each overlapping by 200 bp. Each fragment was cloned into a centromeric plasmid encoding the *URA3* gene. We then transformed the equimolar quantities of these plasmids into the same number of BY4741 cells. In this transformation, we simultaneously transformed YCplac111 (a centromeric plasmid encoding the *LEU2* gene) as a control for transformation efficiency. These transformants were serially diluted (2-fold) and spotted onto SC−Ura and SC−Leu agar plates. As a positive control for replication efficiency, a centromeric plasmid harboring *ARS604* was used. As a negative control, a centromeric plasmid lacking ARS (indicated as "empty") was used. These plates were incubated at 30°C for 3 days, and images were captured using the ChemiDoc Touch Imaging System (Bio-Rad).

#### Pulsed-field gel electrophoresis (PFGE)

Agarose blocks of genomic DNA were prepared using the CHEF Yeast Genomic DNA Plug Kit (Bio-Rad) according to the manufacturer’s instructions with modifications. We used 1 × 10^7^ cells for a plug. Half of each plug was loaded onto a 13 × 14 cm^2^ Certified Megabase agarose gel (Bio-Rad). We used 1% agarose gel in 0.5× TBE to separate elongated chromosome VI (Figure 5D), and 0.8% agarose gel in 1× TAE to separate elongated chromosome IV (Figure 4B). CHEF DNA Size Marker, 0.2–2.2 Mb, *S. cerevisiae* Ladder (Bio-Rad) or CHEF DNA Sized Marker, 1.0–3.1 Mb, *H. wingei* Ladder (Bio-Rad) were used as size markers. All PFGE was performed using CHEF Mapper XA System (Bio-Rad) with a chiller system. CHEF Mapper and running buffer were maintained at 14°C during performing PFGE. The running buffer was the same as the agarose gel buffer. The PFGE conditions were as follows. For separation of elongated chromosome VI (Figure 5D), PFGE was performed in two-state mode at a 120° angle at 6 V/cm for 24 h with switching times ramping 60–120 s. For separation of elongated chromosome IV (Figure 4B), PFGE was performed in two-state mode at a 106° angle at 3 V/cm for 48 h with a fixed switching time of 500 s. Gels were post-stained for 1 h in SYBR Green I (Invitrogen) at room temperature. After staining, images were captured using the ChemiDoc Touch Imaging System (Bio-Rad).

#### Southern blot hybridization

After PFGE to separate elongated chromosome IV and post-staining, we performed Southern blot hybridization. We first incubated the gel in 11 mM HCl for 10 min at room temperature with gentle rotation for depurination, followed by denaturation in 500 mM NaOH and 1.5 M NaCl for 30 min, and then neutralized in the neutralization buffer (500 mM Tris base and 1.5 M NaCl, pH 7.5) for 30 min. We then performed capillary blotting with G Capillary Blotter C-set (TAITEC), transferring DNA to Hybond-N+ hybridization membrane (Cytiva) overnight according to the manufacturer’s instructions. The transferred DNA was crosslinked to the membrane using UVP Crosslinker (CL-3000, Analytik Jena). The membrane was hybridized with a CDP-star-labeled probe derived from the sequences at 556 kb, 559 kb and 563 kb on chromosome IV (see Table S3 for primers to amplify these sequences) in a hybridization buffer (Cytiva) in a glass bottle in a hybridization oven at 55°C overnight. The probe was prepared with AlkPhos Direct Labeling Module (Cytiva) using the primers listed in the Table S3. The membrane was washed twice with primary wash buffer (2 M urea, 0.1% SDS, 50 mM Na phosphate, 150 mM NaCl, 1 mM MgCl_2_, 4% blocking reagent (Cytiva)) and then washed twice with secondary wash buffer (Cytiva, supplemented with 2 mM MgCl_2_). We then added 1 mL of CDP-Star Detection Reagent (Cytiva) onto the membrane and captured images using ChemiDoc Touch Imaging System (Bio-Rad).

#### PNAmp in HEK293T cells

We constructed the duplication reporter plasmid for HEK293T by assembling the gRNA target sequences (up10 and down12) derived from the budding yeast with PCR fragments encoding *PuroR* amplified from Addgene plasmid #171048,^73^ *mCherry* from #159295,^74^ and *SV40 ori*, *FP*, and *GF* from #13031. To construct the expression plasmid for nCas9 and gRNAs, we inserted synthetic DNAs encoding gRNAs targeting up10 and down12 into the BbsI and BsaI sites of Addgene plasmid #74630,^75^ respectively.

The HEK293T cells were grown in Dulbecco’s modified Eagle medium (DMEM, Gibco 11885084) supplemented with 10% fetal bovine serum (FBS, Gibco) and 100 U/mL penicillin and streptomycin (Gibco 15140148) on collagen-coated 12-wells plates (Corning) in an incubator set at 37°C and 5% CO_2_. Plasmid transfection was performed using Lipofectamine 3000 Reagent (Thermo Fisher L3000001). We used 2.5 μg of each plasmid (the reporter plasmid and the gRNA+nCas9 plasmid) for the transfection of the cells in each well of the 12-well plates.

Microscopic images were acquired 72 h after transfection using an inverted microscope Ti-E (Nikon Instruments Inc.) equipped with a sCMOS camera ORCA Fusion-BT (Hamamatsu Photonics). Image acquisition processes were controlled by the software NIS-Elements version 5.3 (Nikon). For the fluorescence images, background signal subtraction was performed with the following settings: the rolling ball radius was set to 20 pixels without smoothing. After background subtraction, the mCherry images were binarized with an intensity threshold of 2,000, despeckled four times, and segmented into regions using the watershed algorithm. In the binarized mCherry images, particles within the area range of 25 to 5,000 pixels were defined as mCherry-positive cells. The total number of these cells was counted, and the fluorescence intensity of each cell was quantified. These binarized mCherry images were then converted into masks, which were applied to the EGFP images to quantify the EGFP fluorescence intensity for each cell. This image processing protocol was applied to cells transfected with plasmids containing only the mCherry gene (EGFP-negative control cells). For each cell, the ratio of EGFP fluorescence intensity to mCherry fluorescence intensity was calculated. For the sample cells, those with a ratio of EGFP fluorescence intensity/mCherry fluorescence intensity exceeding 0.06 were categorized as EGFP-positive cells, and their total number was counted.

After 96 h of transfection, EGFP-positive cells were sorted using BD FACSAria Fusion cell sorter (BD Biosciences) and total DNA containing the plasmids was extracted using Quick-DNA Microprep Kit (ZYMO RESEARCH D3020). To obtain the reporter plasmids, the extracted DNA was transformed into DH5α high Champion cells and ∼500 clones were harvested, then these clones were cultured individually in 96 deep well plates in 300 μL Plusgrow II (Nacalai tesque) supplemented with 100 μg/mL ampicillin (Nacalai tesque) for 24 h. Finally, 100 μL of each culture was harvested and pooled, and the plasmids were extracted using FavorPrep Plasmid Extraction Mini Kit (FAVORGEN). The extracted plasmids were digested with NruI-HF (NEB) for selective linearization of the reporter plasmids (note that the nCas9+gRNA plasmid has no NruI-HF site) and used for library preparation using SQK-NBD114.96 (Oxford Nanopore Technologies), followed by nanopore sequencing using Flongle flow cell (Oxford Nanopore Technologies) on the MinION sequencer. From the generated fastq files, we identified the reads covering the whole plasmids sequences using the 156-bp sequence downstream of NruI cut site and the 122-bp sequence upstream of the cut site as queries in minialign. Next, the reads containing the reconstituted *EGFP* sequence were selected using blast.^76^ To confirm the segmental duplication of the segment flanked by *FP* and *EGF*, these selected reads were used as the first input sequence for YASS. As a second input, we used the reference sequence of the target segment flanked by *FP* and *EGF*.

### Quantification and statistical analysis

Dunnett’s test and Student’s t test were employed to calculate *p* values, as indicated in the figure legends. In general, results were considered statistically significant when *p* < 0.01.

## Supplemental information

Document S1. Figures S1–S7

Table S1. Yeast strains used in this study, related to STAR Methods and Figures 1–6, S1–S3, and S5–S7

Table S2. Plasmids used in this study, related to STAR Methods and Figures 1–7, S2, S3, and S5–S7

Table S3. Oligo DNA used in this study, related to STAR Methods and Figures 1–7, S2, S3, and S5–S7

Table S4. Custom reference genome for Pu-seq, related to Figures 3D, 3E, and S3A

Table S5. Genomic coordinates of chromosome IV in 5 constructed strains, related to Figures 4B and S7F

Table S6. Genomic coordinates of chromosomes IV and VI in 2 constructed strains, related to Figures 5E and 6F

Document S2. Article plus supplemental information
