# Supplementary material for: Strategic targeting of Cas9 nickase induces large segmental duplications
Source: Cell Genom. 2024 Jul 24;4(8):100610. doi: 10.1016/j.xgen.2024.100610 (PMC11406185; doi:10.1016/j.xgen.2024.100610)
Supplement: Document S1. Figures S1–S7 [file mmc1.pdf]

Cell Genomics, Volume *4*

Supplemental information

Strategic targeting of Cas9 nickase induces large segmental duplications

Yuki Sugiyama, Satoshi Okada, Yasukazu Daigaku, Emiko Kusumoto, and Takashi Ito

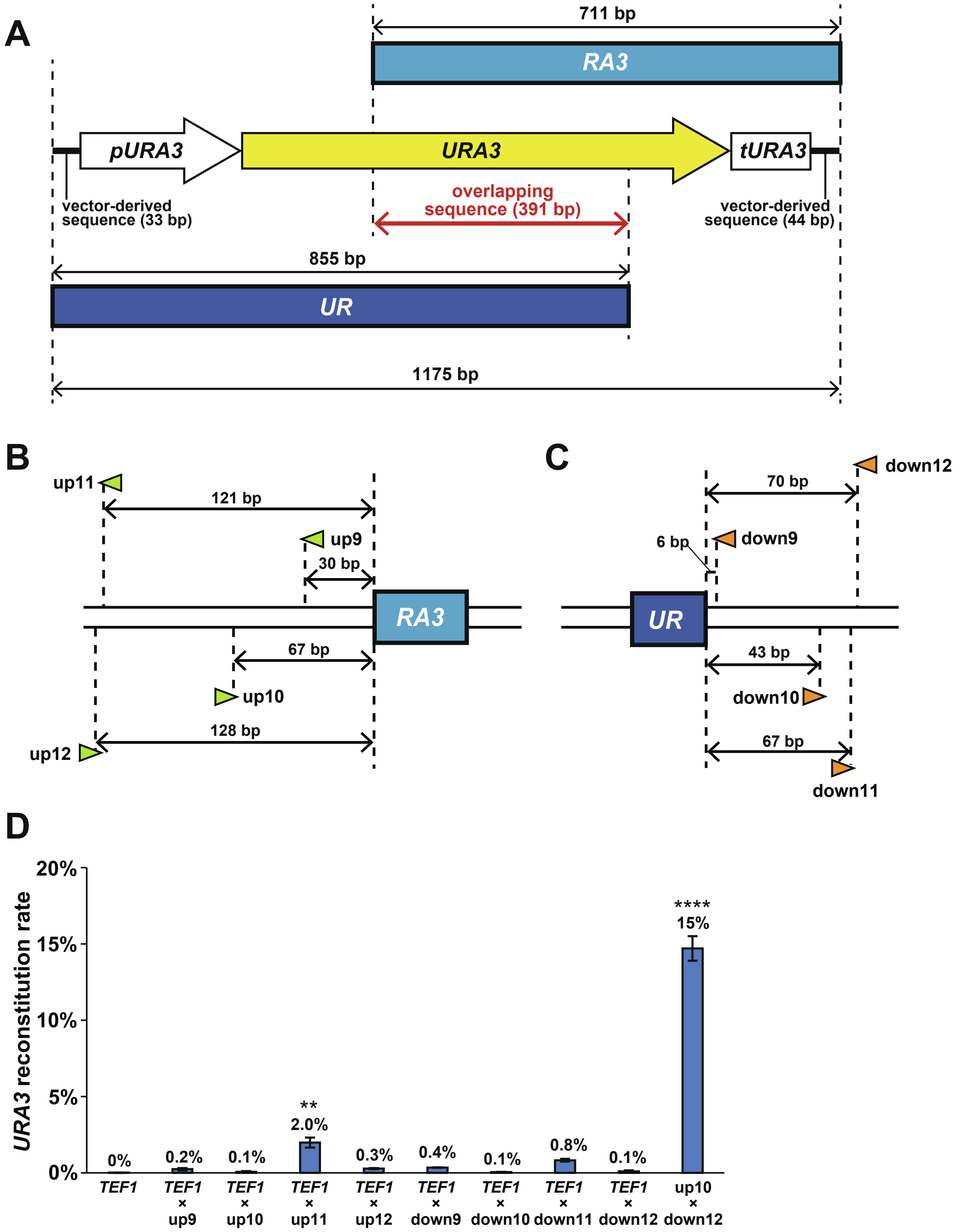

Figure S1. Details of the duplication reporter and gRNAs, related to Figure 1.

(A) Schematic of the *RA3* and *UR* fragments. *RA3* (light blue icon) and *UR* (dark blue icon) are the fragments derived from the *URA3* gene, harboring a 391-bp overlapping sequence *R*. *pURA3* (white arrow) and *tURA3* (white rectangle) indicate the promoter and the terminator of the *URA3* gene, respectively. *URA3* (yellow arrow) indicates the open reading frame of the *URA3* gene.

(B, C) Schematic of the gRNA target positions. Similar to (A), *RA3* (light blue icon) and *UR* (dark blue icon) indicate the *RA3* and *UR* fragments derived from the *URA3* gene. Green and orange arrowheads indicate the gRNA target positions in the upstream of *RA3* (B) and the downstream of *UR* (C), respectively. An arrowhead pointing to the left means that the gRNA sequence is designed on the bottom strand with its PAM: nCas9 (D10A) cleaves the top strand to which the gRNA hybridizes. An arrowhead pointing to the right means that the gRNA target is designed on the top strand with its PAM: nCas9 (D10A) cleaves the bottom strand to which the gRNAs hybridize. For each target site, the distance between its cleavage site and the proximal end of the *RA3* (B) or *UR* (C) is shown.

(D) *URA3* reconstitution rates of strains expressing various gRNAs. Similar to Figure 1C, but each of the gRNAs targeting up9–11 and down 9–11 was paired with *TEF1* gRNA. Error bar, SEM (n = 3). Statistical significance was examined using Dunnett's test (**P < 0.01; ***P < 0.001).

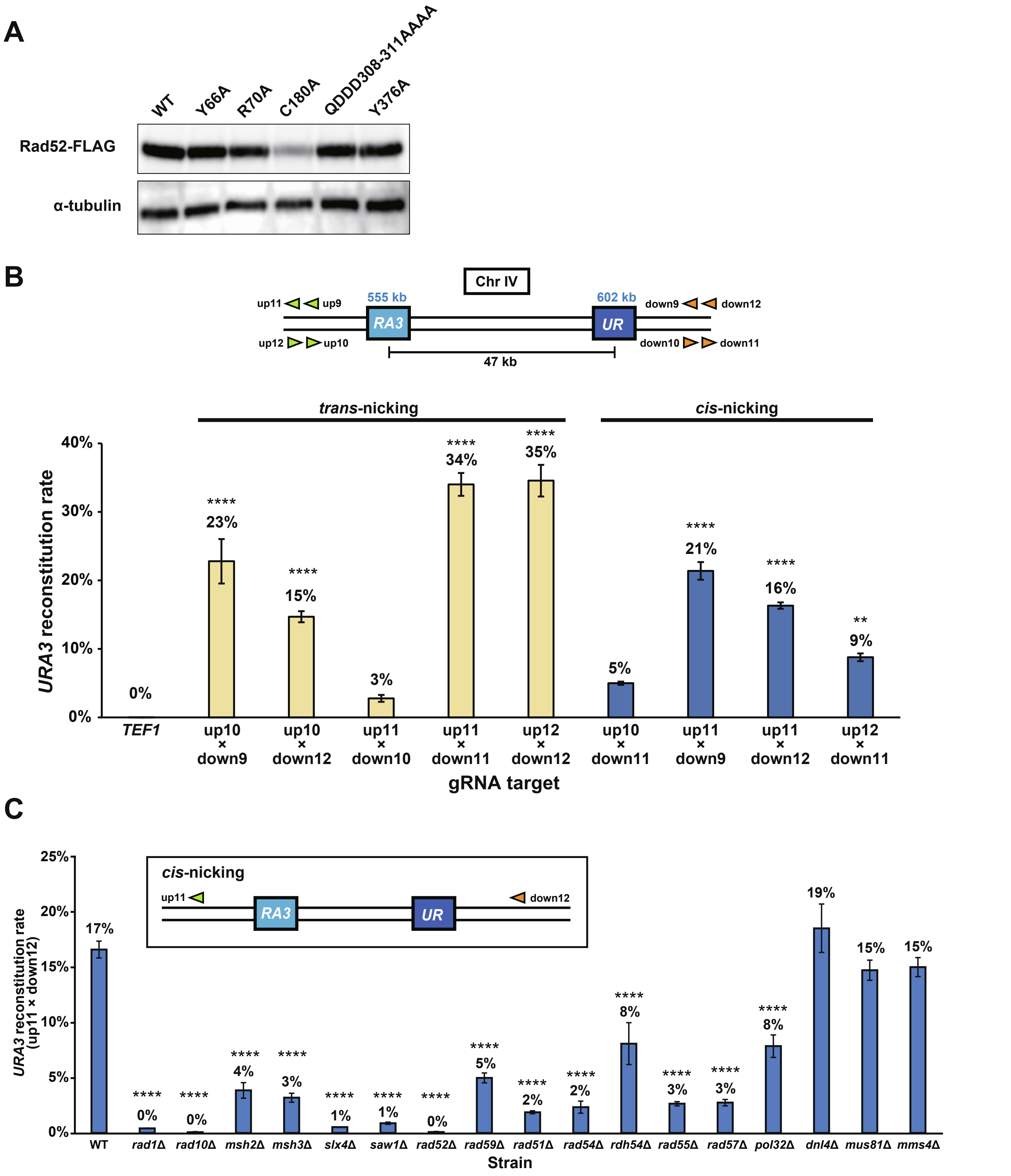

Figure S2. Genetic analysis of PNAmp with trans-nicking and cis-nicking, related to Figure 2.

(A) Western blotting of Rad52 proteins expressed in the *rad52*Δ strains with the indicated alleles. Rad52 proteins were C-terminally FLAG-tagged and detected with anti-FLAG antibody. Tubulin-α was used as a loading control for each sample.

(B) PNAmp with various gRNA pairs. A schematic of the genetic reporter system on chromosome IV with the positions of gRNA targets (top). The *URA3* reconstitution rates for the indicated gRNA pairs (bottom). Yellow bars indicate PNAmp with trans-nicking, and blue bars indicate PNAmp with cis-nicking. Error bars indicate SEM (n = 3). The statistical significance was examined between the sample strains and the control strain expressing gRNA targeting *TEF1* using Dunnett's test (**P < 0.01; ****P < 0.0001).

(C) Effects of deleting genes related to DNA repair on non-canonical PNAmp with cis-nicking by gRNAs targeting up11 and down12. Error bars indicate SEM (n = 3). The statistical significance between the mutant strains and the WT strain was examined using Dunnett's test (****P < 0.0001).

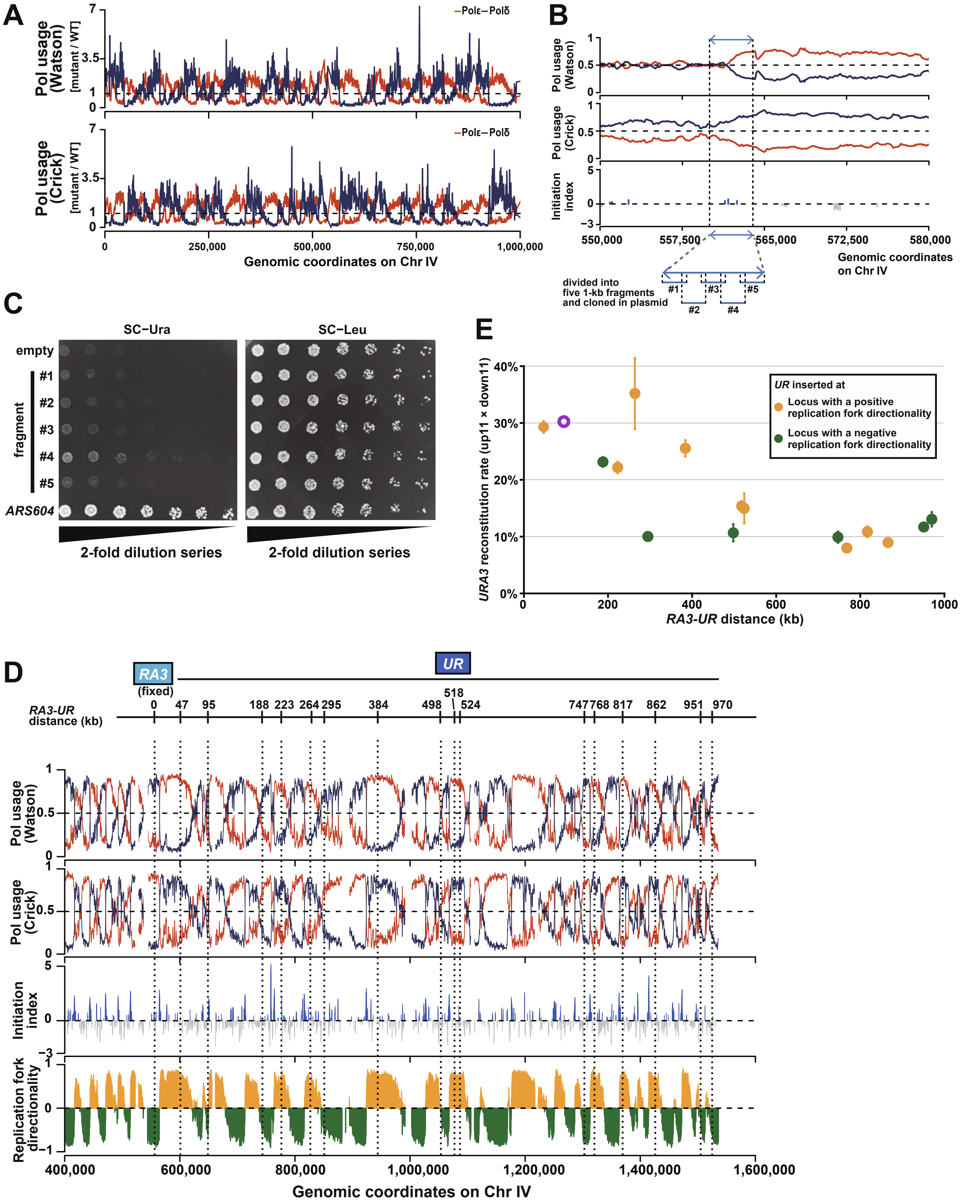

(legend on next page)

Figure S3. Effects of replication initiation from inside the target segment and RFD on PNAmp, related to Figures 3 and 4.
(A) Relative polymerase usage revealed by Pu-seq. Note that the ribonucleotide incorporation of the polymerase mutant strains is normalized to that of the strain encoding the wild-type polymerase. A reciprocal pattern appears between Polε and Polδ on each strand. Similarly, a reciprocal pattern appears between the Watson and Crick strands for each polymerase. Data were shown the region between positions 1 and 1,000,000 of chromosome IV. Note that the genomic coordinates are shifted compared to the standard reference sequence of the S288C strain.
(B) Pu-seq patterns between positions 550 kb and 580 kb of the *ars418Δ ars419Δ* strain. Weak peaks of initiation index were found around 562 kb. Blue arrows indicate the region spanning the weak initiation index peaks. At the bottom, a schematic of the region divided into five 1-kb fragments is shown. These fragments were used in the replication activity assay in (C).
(C) Spot test assay of the replication activity of the five 1-kb fragments in (B). The same number of the wild-type cells were co-transformed with the centromeric plasmids harboring the *URA3* gene and the indicated fragments and the YCplac111, a *LEU2*-marked centromeric plasmid, as a normalizer of the transformation efficiency. Transformants are serially diluted and spotted on SC−Ura and SC−Leu agar plates and incubated at 30°C for 3 days. Note that the cells transformed with the plasmid carrying the fragment #4 showed better growth than the others, suggesting that it has an activity to induce plasmid replication.
(D) Pu-seq and RFD pattern of chromosome IV in the wild-type PNAmp strain. The first and second tracks show the polymerase usage on the Watson and Crick strands, respectively. The third track shows the initiation index (blue and gray bars). The fourth track shows the RFD, with green and orange bars indicating the dominance of the leftward and rightward forks, respectively. The frequency of being replicated by the rightward fork exceeds that by the leftward fork at a locus with positive RFD value (orange), and vice versa at locus with negative RFD value (green). Note that the genomic coordinates of chromosome IV in the PNAmp strains are shifted by ~10 kb compared to the standard reference sequence of the S288C strain because the *HO* locus carries the nCas9 expressing cassette.
(E) Effects of target size and RFD on PNAmp. Similar to Figure 4A, but each of the 16 strains is colored according to the estimated RFD at its *UR*, which was estimated from the RFD at the corresponding positions in the parental reporter strain in which the distance between *RA3* and *UR* is 47 kb. Orange and green dots indicate that the estimated RFD values are positive and negative, respectively. The open dot indicates the strain carrying UR at 650 kb, in which the distance between *RA3* and *UR* is 95 kb. In this strain, the RFD at *UR* cannot be estimated from the RFD at the corresponding position in the parental strain, because the *UR* disrupted *ARS421* located very close to the insertion site, thereby severely perturbing the replication status in its vicinity. Error bars indicate SEM (n = 3).

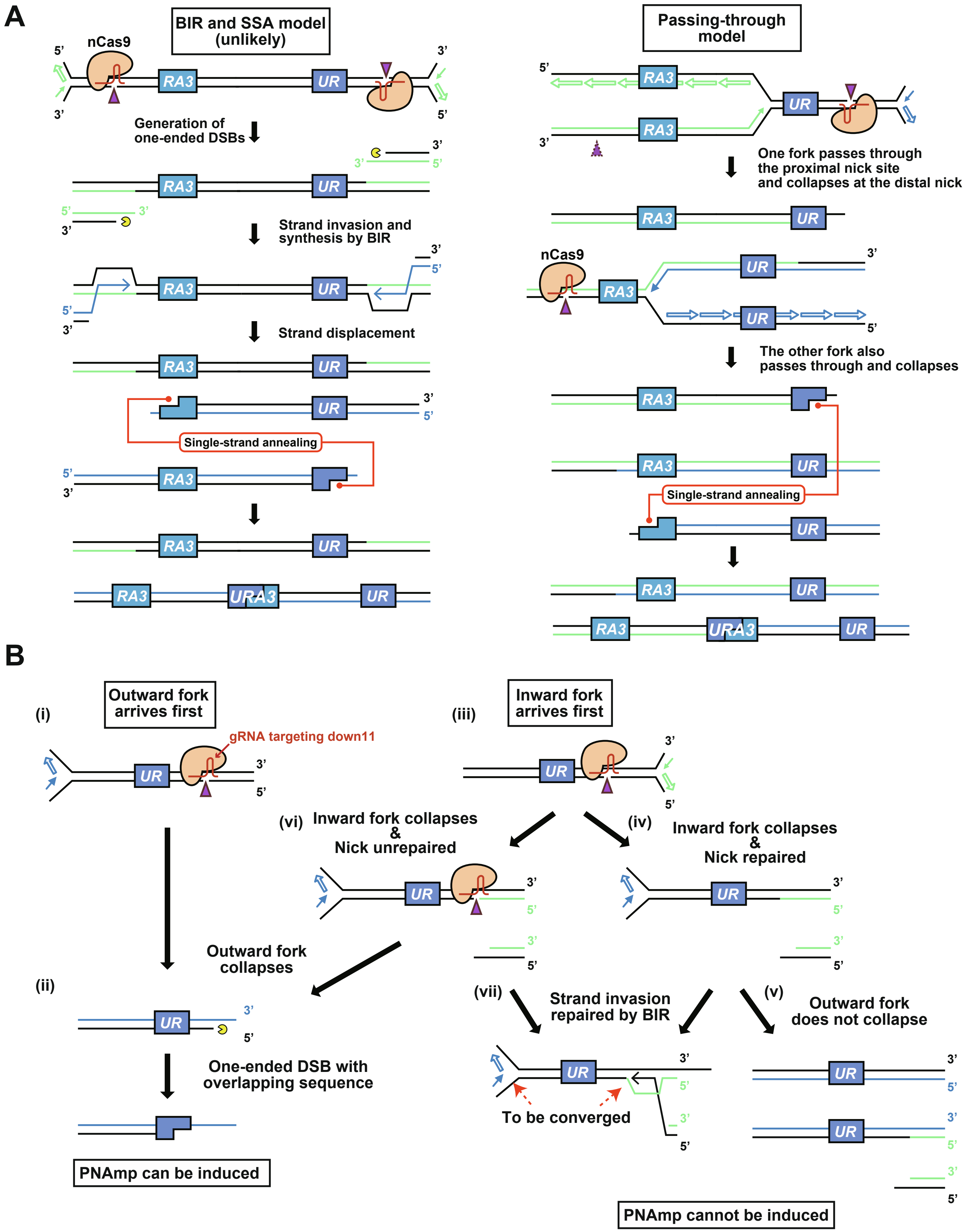

(legend on next page)

Figure S4. Models for PNAmp under non-ideal conditions, related to Figures 3 and 4.

(A) Models for PNAmp without replication initiation from inside the target segment. (Left) Double BIR model. The replication forks approaching from outside the target segment (inward forks) collapse at the nicks. The 3′-overhang ssDNAs derived from the one-ended DSBs invade their allelic positions on the sister chromatids to initiate BIR (double BIR). If the two BIR forks pass each other to reach the nicks at the opposite sides of the target segment, they will collapse to generate a pair of one-ended DSBs that can mediate SSA to duplicate the target segment. However, the PNAmp in the *ars418*Δ *ars419*Δ strain was unlikely to use this mechanism because it occurred even in the absence of *RAD51* and *POL32* required for BIR. (Right) Passing-through model. Suppose that nCas9 dissociates from its target site upstream of *RA3* to allow nick sealing before the rightward fork arrives. In such a case, the rightward fork will pass through the proximal, sealed nick site upstream of *RA3* and reach at the distal nick downstream of *UR* to collapse there. This event leads to the generation of two chromatids, one truncated at the one-ended DSB and the other connected to the yet-to-be-replicated parental DNA. If the latter DNA escapes re-nicking at the target site downstream of *UR*, the leftward fork will pass through it and reach at the opposite end of the target segment. If the nick is introduced before the arrival of the leftward fork, it will collapse there to generate a second one-ended DSB. These two one-ended DSBs share *R* to mediate SSA to duplicate the target segment independently of *RAD51* and *POL32*. Note that the SD occurs on one chromatid but not on the other in this case. Purple arrowheads indicate the positions of nicks induced by nCas9. Solid arrows (blue and green) indicate leading strands, and open arrows (blue and green) indicate lagging strands.

(B) Models of PNAmp under undesirable RFD. The schematic shows only the downstream region of *UR*, with the nick being introduced to the bottom strand with the gRNA targeting down11. Suppose that the outward (rightward) fork arrives at the nick earlier than the inward (leftward) fork (i). In this case, the outward fork will collapse to generate a one-ended DSB harboring the *R* to mediate SSA for PNAmp (ii). Conversely, suppose that the inward (leftward) fork arrives earlier than the outward (rightward) fork (iii). In this case, the inward fork will collapse to generate a one-ended DSB not harboring the *R* to mediate SSA for PNAmp. Note that this event generates two sister chromatids, one truncated by the one-ended DSB and the other connected to the parental DNA to be replicated by the rightward fork (iv & vi). If the latter chromatid escapes re-nicking by the nCas9 or subject to nick sealing before the outward (rightward) fork arrives (iv), the fork will pass through the nCas9 target site, not generating a one-ended DSB carrying the *R* (v). In contrast, if the latter chromatid is re-nicked before the outward (rightward) fork arrives (vi), the fork will collapse at the nCas9 target site, generating a one-ended DBS carrying the *R* to mediate SSA for PNAmp (ii). These cases may result in the trisomy of the sequence downstream of the initial nick site (v and vi). It is also possible that the BIR fork derived from the one-ended DSB generated by the collapse of the inward (leftward) fork merges with the outward (rightward) fork to accomplish the repair process correctly or without generating the segmental duplication (vii). Purple arrowheads indicate the positions of nicks induced by nCas9. The inward and outward forks are colored green and blue, respectively. Solid arrows indicate leading strands, and open arrows indicate lagging strands. *RA3* and *UR* indicate the fragments derived from the *URA3* gene harboring the overlapping sequence.

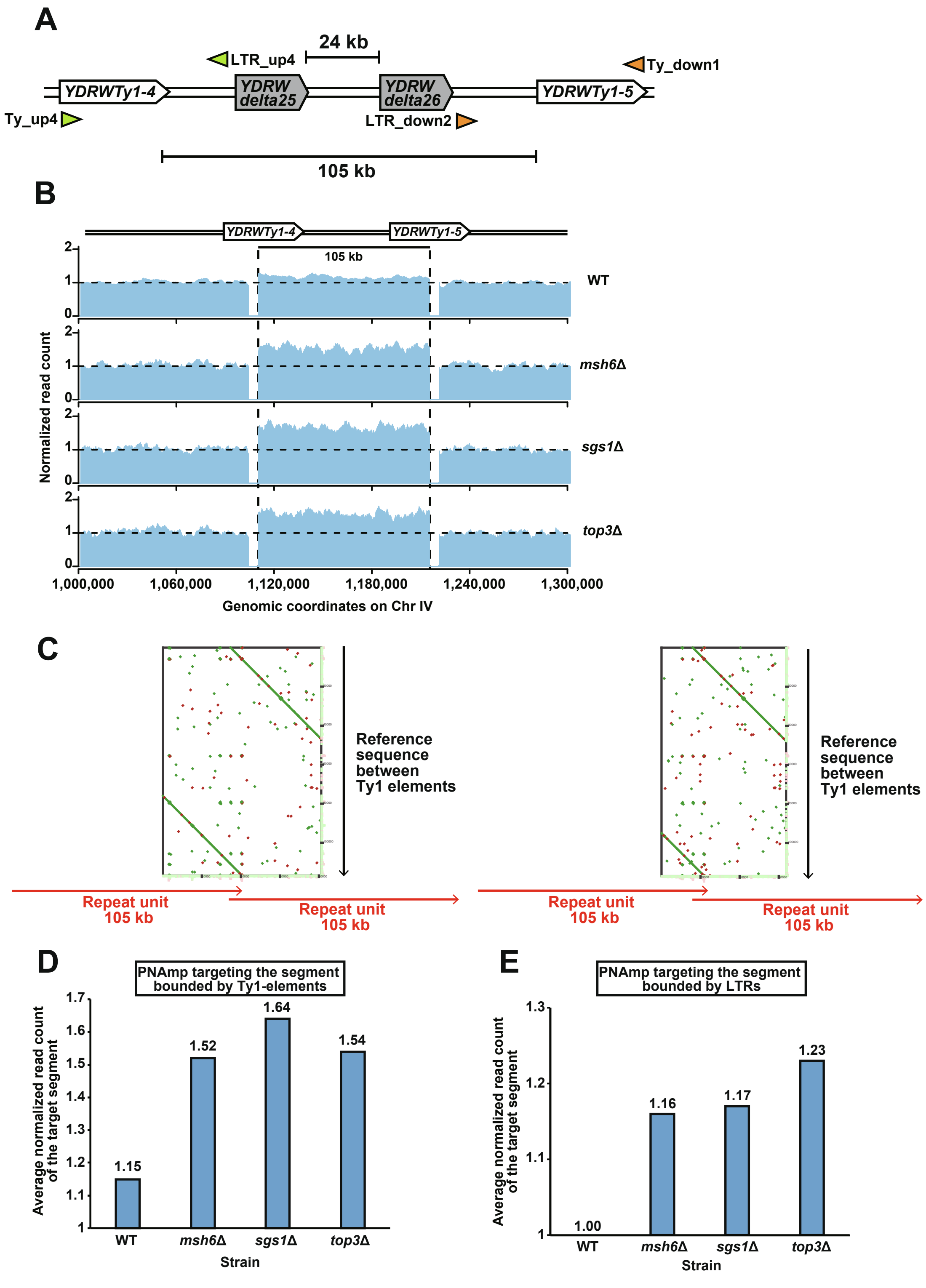

Figure S5. PNAmp in natural genomic contexts, related to Figure 4.

(A) Schematic of PNAmp using naturally occurring repetitive elements. White and gray arrows represent Ty1 elements separated by 105 kb (*YDRWTy1-4* and *YDRWTy1-5*) and LTRs separated by 24 kb (*YDRWdelta25* and *YDRWdelta26*) on chromosome IV, respectively. Green and orange arrowheads indicate the positions of gRNA target sequences located upstream and downstream of the repetitive sequences, respectively.

(B) Normalized read counts of the target segments in whole-genome nanopore sequencing of pooled colonies. A schematic depicting the target segment bound by the two Ty1 elements is shown at the top. The genotypes of the sequenced strains are displayed to the right of the tracks. Note that Ty1 elements were masked in the customized reference genome sequence used for mapping, resulting in the gaps of the read counts.

(C) Dot plot analysis of nanopore reads spanning the duplication junction. Of the 6,955 reads over 90 kb obtained by the sequencing of pooled wild-type colonies in (B), 20 contained the junction between the tail and head portions of the 105-kb target segment.

(D) Normalized read count of the 105-kb target segment between two Ty1 elements in strains proficient and deficient in MMR. We compared the average of normalized read counts throughout the 105-kb segments among the wild-type (WT) and the three MMR mutants.

(E) As in (D), but for the 24-kb segment between the two LTRs. Note that the duplication was not detectable in the wild-type strain.

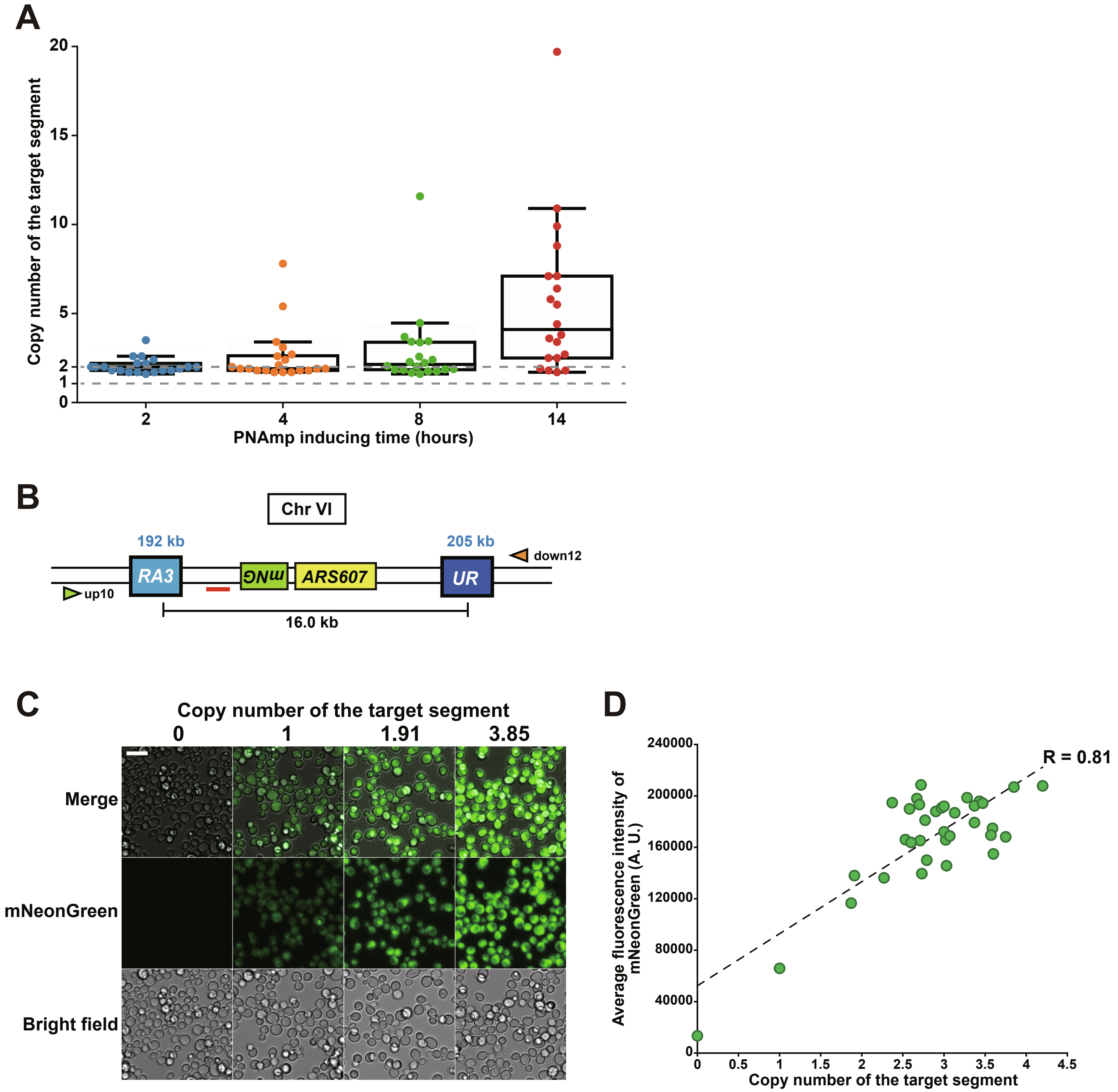

Figure S6. Time-course and gene dosage analysis of iterative PNAmp, related to Figure 5.
(A) Target copy number along with the induction of PNAmp. We induced PNAmp of the 12.9-kb fragment on chromosome VI using gRNA pairs targeting up10 and donw12 (Figure 5A), isolated 20 Ura^+ clones at each time point, and examined the target copy number in each clone by qPCR.
(B) Schematic of the target segment containing the *mNeonGreen* gene on chromosome VI. As in Figure 5A, but the insertion of the *mNeonGreen* gene cassette (*mNG*) increased the target size from 12.9 kb to 16.0 kb.
(C) Microscopic images of the Ura^+ clones obtained by inducing PNAmp. The numbers above the panels indicate the target copy number determined by qPCR using the amplicon shown in (B). The zero-copy number indicates that the clone does not contain the *mNeonGreen* gene, serving as a negative control. Scale bar, 10 µm.
(D) Correlation between the average fluorescence intensity and the target copy number. A.U.: arbitrary unit, R: Pearson's correlation coefficient.

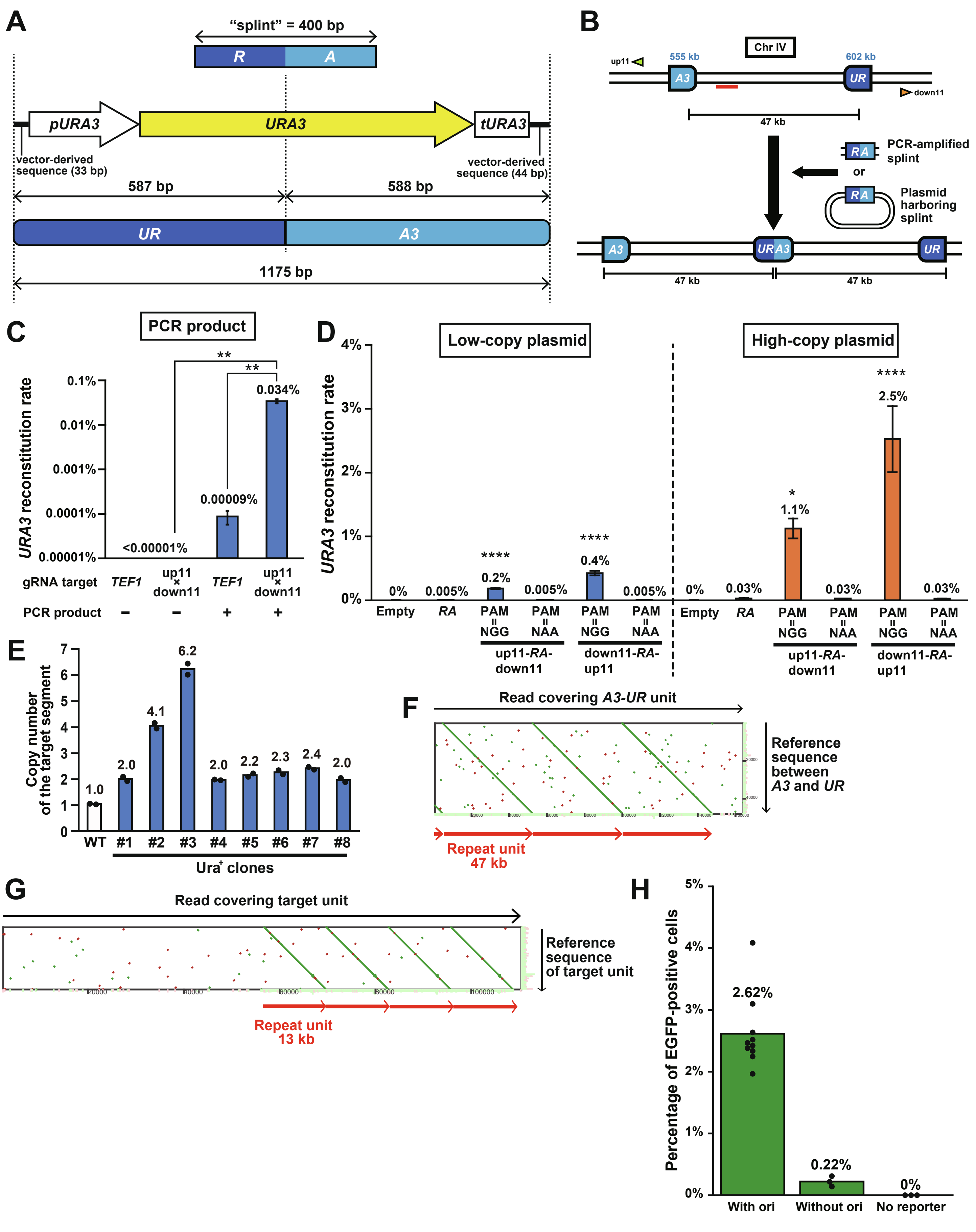

(legend on next page)

Figure S7. Splinted PNAmp and PNAmp in HEK293T cells, related to Figures 6 and 7.

(A) Schematic of the *UR* and *A3* fragment, sharing no overlapping sequences. *pURA3* and *tURA3* indicate the promoter and the terminator of the *URA3* gene, respectively. *URA3* colored yellow indicates the open reading frame of the *URA3* gene. *UR* colored dark blue and *A3* colored light blue indicate the fragments derived from the *URA3* gene, respectively. *RA* shown at the top indicates the splint sequence, spanning the boundary of the *UR* and *A3* fragments, which was used as a PCR product or carried on the plasmids.

(B) Schematic of PNAmp with a splint DNA for the 47-kb target segment bounded by *A3* and *UR*. Two fragments derived from the *URA3* gene (*A3* colored light blue and *UR* colored dark blue) harboring no overlapping sequence were inserted at the interval of 47 kb on chromosome IV. The cells carrying this genetic reporter were transformed with a splint PCR product or a plasmid harboring the splint sequence prior to Dox-induced expression of nCas9 and gRNAs. The cells were plated on SC or SC−Ura medium to evaluate the *URA3* reconstitution rate. Note that the inserted positions of *A3* and *UR* is indicated in genomic coordinates in chromosome IV of the PNAmp strains carrying the nCas9 expressing cassette in the *HO* locus in chromosome IV. In this genomic coordinate, the sequence downstream of *HO* is shifted by ~10 kb compared to the standard reference sequence of the S288C strain.

(C) PNAmp with a splint DNA prepared by PCR. *URA3* reconstitution rates are shown for strains expressing the indicated gRNAs with and without PCR product transformation. Error bars indicate SEM (n = 3). The rates for the samples without PCR product transformation are denoted as <0.00001% because no Ura^+ colonies appeared even though more than 2×10^7 colony forming units were plated on the SC−Ura plates. Statistical significance was examined between the strains with and without PCR product transformation, both expressing gRNAs targeting up11 and down11, using Student's *t*-test (**P < 0.01). Similarly, statistical significance was examined between the strains expressing gRNAs targeting up11 and down11 and gRNA targeting *TEF1*, both transformed with the PCR product, using Student's *t*-test (**P < 0.01).

(D) PNAmp with splint plasmids. The *URA3* reconstitution rate of the strains carrying the indicated splint plasmids. Error bars indicate SEM (n = 3). The statistical significance between the strains carrying the splint and empty plasmid was examined using Dunnett's test (*P < 0.05, ****P < 0.0001).

(E) Copy number of the target segment in eight Ura^+ colonies determined by qPCR. These colonies were obtained by PNAmp with a high-copy splint plasmid in (D) (down11-RA-up11, PAM = NGG). Dots indicate technical replicates for each clone. The position of the amplicon used for qPCR is shown as a red bar in (B).

(F) Dot plot between a representative nanopore read obtained from the clone #2 on the SC−Ura plate in (E) and the reference sequence of the 47-kb segment. Each red arrow indicates the single unit of the 47-kb segment. Of the 470 reads over 150 kb, two indicated the presence of at least four copies of the target segment.

(G) Dot plot between a representative nanopore read obtained from the clone indicated in Figure 6H and the reference sequence of the 13-kb target segment. Of the 27,475 reads over 30 kb, three contained the entire amplicon to prove tandem quadruplication of the target segment.

(H) Frequency of EGFP-positive cells among mCherry-positive cells. The percentages of EGFP-positive cells were shown for HEK293T cells transformed with the reporter plasmid harboring SV40 ori, a reporter plasmid derivative lacking SV40 ori, and no reporter plasmid. Since no fluorescence was observed in the absence of *EGFP*-derived fragments (no reporter), the effects of autofluorescence difficult to separate from EGFP, if any, were negligible.

Supplemental references

[S1] Brachmann, C.B., Davies, A., Cost, G.J., Caputo, E., Li, J., Hieter, P., and Boeke, J.D. (1998). Designer deletion strains derived from *Saccharomyces cerevisiae* S288C: a useful set of strains and plasmids for PCR-mediated gene disruption and other applications. Yeast *14*, 115–132. https://doi.org/10.1002/(SICI)1097-0061(19980130)14:2<115::AID-YEA204>3.0.CO;2-2

[S2] Gietz, R. D., and Sugino, A. (1988). New yeast-*Escherichia coli* shuttle vectors constructed with in vitro mutagenized yeast genes lacking six-base pair restriction sites. Gene *74*, 527–534. https://doi.org/10.1016/0378-1119(88)90185-0
